# Endothelial adenosine A2a receptor-mediated glycolysis is essential for pathological retinal angiogenesis

**DOI:** 10.1038/s41467-017-00551-2

**Published:** 2017-09-19

**Authors:** Zhiping Liu, Siyuan Yan, Jiaojiao Wang, Yiming Xu, Yong Wang, Shuya Zhang, Xizhen Xu, Qiuhua Yang, Xianqiu Zeng, Yaqi Zhou, Xuejiao Gu, Sarah Lu, Zhongjie Fu, David J. Fulton, Neal L. Weintraub, Ruth B. Caldwell, Wenbo Zhang, Chaodong Wu, Xiao-Ling Liu, Jiang-Fan Chen, Aftab Ahmad, Ismail Kaddour-Djebbar, Mohamed Al-Shabrawey, Qinkai Li, Xuejun Jiang, Ye Sun, Akrit Sodhi, Lois Smith, Mei Hong, Yuqing Huo

**Affiliations:** 1 Drug Discovery Center, Key Laboratory of Chemical Genomics, Peking University Shenzhen Graduate School, Shenzhen, 518055 China; 2 Vascular Biology Center, Department of Cellular Biology and Anatomy, Medical College of Georgia, Augusta University, Augusta, GA 30912 USA; 3 State Key Laboratory of Mycology, Institute of Microbiology, Chinese Academy of Science, Beijing, 100101 China; 4 Molecular Neuropharmacology Laboratory, School of Optometry and Ophthalmology and Eye Hospital, Wenzhou Medical University, Wenzhou, 325035 China; 5 Department of Ophthalmology, Boston Children’s Hospital, Harvard Medical School, Boston, MA 02115 USA; 6 Department of Ophthalmology & Visual Sciences, University of Texas Medical Branch (UTMB), Galveston, TX 77555 USA; 7 Department of Nutrition and Food Science, Texas A&M University, College Station, TX 77843 USA; 8 Department of Neurology, Boston University School of Medicine, Boston, MA 02118 USA; 9 Department of Anesthesiology and Perioperative Medicine, University of Alabama at Birmingham, Birmingham, AL 35249 USA; 10 Department of Physiology, Medical College of Georgia, Augusta University, Augusta, GA 30912 USA; 11 James and Jean Culver Vision Discovery Institute, Medical College of Georgia, Augusta University, Augusta, GA 30912 USA; 12 Wilmer Eye Institute, Johns Hopkins School of Medicine, Baltimore, MD 21287 USA

## Abstract

Adenosine/adenosine receptor-mediated signaling has been implicated in the development of various ischemic diseases, including ischemic retinopathies. Here, we show that the adenosine A2a receptor (ADORA2A) promotes hypoxia-inducible transcription factor-1 (HIF-1)-dependent endothelial cell glycolysis, which is crucial for pathological angiogenesis in proliferative retinopathies. Adora2a expression is markedly increased in the retina of mice with oxygen-induced retinopathy (OIR). Endothelial cell-specific, but not macrophage-specific *Adora2a* deletion decreases key glycolytic enzymes and reduces pathological neovascularization in the OIR mice. In human primary retinal microvascular endothelial cells, hypoxia induces the expression of ADORA2A by activating HIF-2α. ADORA2A knockdown decreases hypoxia-induced glycolytic enzyme expression, glycolytic flux, and endothelial cell proliferation, sprouting and tubule formation. Mechanistically, ADORA2A activation promotes the transcriptional induction of glycolytic enzymes via ERK- and Akt-dependent translational activation of HIF-1α protein. Taken together, these findings advance translation of ADORA2A as a therapeutic target in the treatment of proliferative retinopathies and other diseases dependent on pathological angiogenesis.

## Abstract (web summary)

Pathological angiogenesis in the retina is a major cause of blindness. Here the authors show that adenosine receptor A2A drives pathological angiogenesis in the oxygen-induced retinopathy mouse model by promoting glycolysis in endothelial cells via the ERK/Akt/HIF-1α pathway, thereby suggesting new therapeutic targets for disease treatment.

## Introduction

Pathological angiogenesis is among the most common causes of irreversible blindness for individuals at all ages, including newborns (retinopathy of prematurity), middle-age adults (proliferative diabetic retinopathy, PDR) and the elderly (age-related macular degeneration, AMD). Retinal neovascularization is one of the major pathologies for these sight-threatening retinopathies^1–3^. Neovascular tissues are characterized by incompetent, leaky blood vessels that can bleed or contract, leading to hemorrhage or retinal detachment and eventually to blindness^1^. Increased endothelial sprouting and proliferation are major cellular events causing pathological proliferative retinopathies^4, 5^. Therefore, deciphering the molecular mechanisms underlying these early cellular events is key to understanding and further developing novel therapeutic approaches for the prevention or treatment of these vision-threatening diseases.

Increased emerging evidence indicates that not only signals from growth factors and the Notch pathway, but also glucose metabolism, control endothelial cell (EC) proliferation, migration, and neovascularization^6, 7^. ECs rely on glycolysis rather than oxidative metabolism for ATP production and vessel sprouting^8^. Reduction of glycolysis using an inhibitor of 6-phosphofructo-2-kinase/fructose-2, 6-bisphosphatase isoform 3 (PFKFB3) or endothelial-specific genetic deletion of *Pfkfb3* inhibits pathological angiogenesis in murine models of AMD and oxygen-induced retinopathy (OIR), respectively^9, 10^. Importantly, increased glycolysis, evidenced by an increased level of lactate in vitreous fluid, has been demonstrated in patients with PDR^11^. Due to this close association between EC glycolysis and pathological retinal angiogenesis as well as substantial demand for new treatment of retinopathies, it is pressing to uncover practical targeting molecules that regulate the glycolytic pathway in retinal ECs.

Hyperactivation of adenosine signaling has been implicated in cellular responses to hypoxia and the development of various ischemic diseases^12^. Loss of functional vasculature and consequent hypoxia precedes the development of ischemic proliferative retinopathies. Hypoxia results in marked increases in adenosine production and adenosine receptor signaling^12^. Indeed, in a canine model of OIR, peak adenosine levels in the retina correlated temporally with active vasculogenesis in the retina^13^. Immunoreactivity of adenosine A2a receptor (Adora2a), one of the adenosine receptors, is prominent in ECs and angioblasts in newly formed blood vessels, and is significantly elevated in intravitreal neovascularization^14^. Yet it remains unclear whether retinal endothelial adenosine-Adora2a signaling plays a role in glycolysis and pathological retinal angiogenesis, although in mouse models of wound healing and hind limb ischemia, activation of Adora2a brings about beneficial angiogenesis^15, 16^.

In this study, we showed that Adora2a expression is significantly increased in pathological retinal neovessels in OIR. We found that hypoxia upregulates ADORA2A expression by activating hypoxia-inducible transcription factor (HIF)-2α in human microvascular retinal ECs (HRMECs). Using gain- and loss-of-function approaches, we identified ADORA2A as a key regulator of the metabolic and angiogenic switch in HRMECs in vitro. Our study further demonstrated that endothelium-specific *Adora2a* deletion reduces glycolysis and pathological neovascularization in retinopathy in vivo.

## Results

### Expression of Adora2a in retinal pathological angiogenesis

**Fig. 1:**
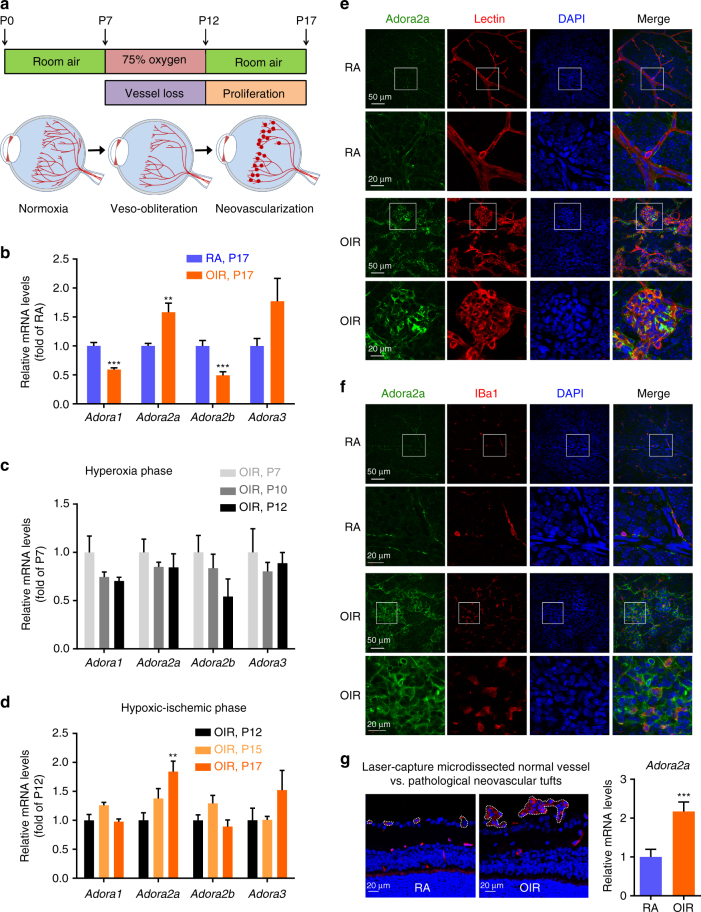
Localization and expression of Adora2a in rodent proliferative retinopathy. **a** Schematic illustration of mouse OIR model. Neonatal mice with nursing mothers were exposed to 75% O_2_ from postnatal day (P) 7 to P12, followed by room air (*RA*) with maximum neovascularization at P17. **b** Real-Time PCR analysis of *Adora1*, *Adora2a*, *Adora2b*, and *Adora3* mRNA expression in the whole retina. Retinas were from RA or OIR mice at P17. ***P < 0.001 vs. RA group (*n* = 7 mice per group). **c**, **d** Real-Time PCR analysis of adenosine receptor mRNA expression in the whole retina. Retinas were obtained from mice at the times indicated. Data were normalized to both the expression of internal control and to gene mRNA expression of each RA control at each time point. **P < 0.01 vs. P12 (*n* = 4 mice per group for **c** and *n* = 7 mice per group for **d**). **e**, **f** Localization and expression of Adora2a in the RA and OIR retinas. Retinopathy was induced in wild-type mice. P17 RA and OIR retinas were stained with Adora2a (*green*), isolectin B4 (Lectin, *red*, vessel, **e**), or IBa1 (*red*, macrophages/microglias, **f**) and DAPI (*blue*, nuclei). In all, *2nd* and *4th* rows are magnification of the *boxed* regions in the *1st* and *3rd* rows, respectively. *Scale bar*: 50 μm (*1st* and *3rd* rows) and 20 μm (*2nd* and *4th* rows). **g** Real-Time PCR analysis of *Adora2a* mRNA expression in laser-capture microdissected pathological neovessels (tufts) from OIR mice compared with normal vessels from control mice raised in RA at P17. ***P < 0.001 vs. RA (*n* = 4 per group). Data are represented as means ± s.e.m. Statistical significance was determined by unpaired Student’s *t*-test (for **b**, **g**) and one-way ANOVA followed by Bonferroni test (for **c**, **d**)

To study the role of adenosine receptors (ADORs) in pathological angiogenesis, we first assessed the expression profile of ADORs in the retinas of a mouse OIR model (Fig. 1a). Real-time PCR analysis revealed that expression of the *Adora2a* gene was significantly increased while adenosine A_1_ receptor (*Adora1*) and adenosine A_2B_ receptor (*Adora2b*) levels were reduced on postnatal day (P)17 in OIR retinas, compared with controls in room air (RA) (Fig. 1b). We next performed a time-course analysis of messenger RNA (mRNA) levels of *Adors* from P7 to P12 (the hyperoxia phase), and P12 to p17 (the hypoxic-ischemic phase) of OIR retinas. We found no noticeable changes in the expression of *Adors* from P7 to P12 (Fig. 1c), whereas expression of *Adora2a* steadily increased from P12 to P17 (Fig. 1d), indicative of a sustained increase in the expression of *Adora2a* throughout the hypoxic-ischemic phase of OIR. To localize the expression of Adora2a, we performed double-immunofluorescence staining of whole-mount retinas from OIR or control mice using a well-characterized monoclonal antibody for Adora2a^17, 18^, and a retinal blood vessel marker (Isolectin B4), or a macrophage/microglia marker (IBa1). In RA control retinas, Adora2a was present in the blood vessel wall, whereas in OIR retinas, Adora2a was strongly expressed within and around pathological neovascular tufts, particularly in and around ECs and macrophages/microglias, as indicated by its colocalization with blood vessels and IBa1 (Fig. 1e, f). Ablation of Adora2a expression in retinas of global homozygous *Adora2a* knockout mice (*Adora2a* ^*−/−*^, Supplementary Fig. 1) confirmed antibody specificity. Using laser-capture microdissection to isolate pathological neovascular tufts from OIR mice and normal vessels from control (RA) mice, we confirmed that *Adora2a* mRNA level is higher in retinal neovessels compared with normal vessels (Fig. 1g). Importantly, type 1 diabetic patients homozygous for the T allele of *ADORA2A* SNP rs2236624 and rs4822489, two genotypes associated with low incidence of PDR^19^, had lower levels of *ADORA2A* mRNA compared with their controls, which are type 1 diabetic patients homozygous for the C allele of *ADORA2A* SNP rs2236624 and G allele of rs4822489 (Supplementary Fig. 2). These findings indicate a close association of a high level of ADORA2A expression with proliferative retinopathies.

### Retinal neovascularization in mice lacking endothelial Adora2a

**Fig. 2:**
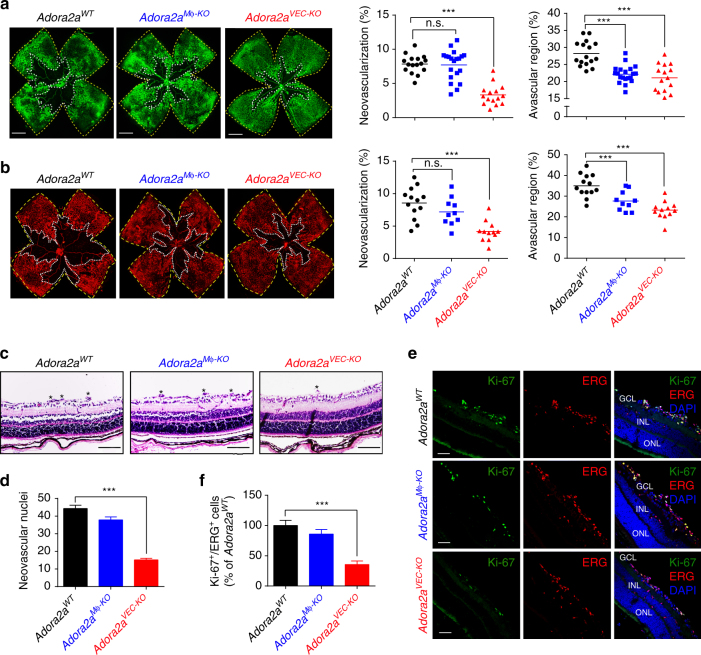
Endothelial *Adora2a* deficiency significantly decreases formation of pathological neovascularization in OIR retinas. **a**, **b** Quantification of pathological neovascularization and vessel dropout area (within the *white borders*) in postnatal day (P)17 OIR retinas. *Adora2a* ^flox/flox^ (*Adora2a* ^*WT*^), *Adora2a* ^flox/flox^Lysm^cre/cre^ (*Adora2a* ^*Mφ-KO*^), and *Adora2a* ^flox/flox^Cdh5^cre^ (*Adora2a* ^*VEC-KO*^) mice were exposed to 70% O_2_ **a** or 75% O_2_ **b**. Areas of pathological neovascularization and vessel dropout are quantified as percentage of total retinal area. *n* = 16, 20, 15 retinas for **a**; *n* = 13, 10, 12 retinas for **b**; ***P < 0.001 vs. *Adora2a* ^*WT*^ group. *Scale bars*: 1000 μm. **c** Histological analysis of infiltration of neovascular nuclei from inner limiting membrane into vitreous in the OIR retinas. Nuclei on the vitreal side of the inner limiting membrane are indicated by *asterisk*. *Scale bars*: 100 μm. **d** Quantitative analysis of the number of neovascular nuclei in the OIR retinas. *P < 0.001 (*n* = 6 mice for *Adora2a* ^*WT*^ group; *n* = 8 for *Adora2a* ^*Mφ-KO*^ and *Adora2a* ^*VEC-KO*^ groups). **e** Ki-67 immunofluorescent staining on OIR retinas. Representative *green* (Ki-67), *red* (ERG), *blue* (nuclei, DAPI), and merged images were captured with confocal fluorescent microscopy. *GCL* ganglion cell layer, *INL* inner nuclear layer, *ONL* outer nuclear layer. *Scale bars*: 50 μm. **f** Quantitative analysis of the Ki-67 and ERG double-positive cells in each group. *P < 0.001 (*n* = 9 mice for each group). Data are represented as means ± s.e.m. Statistical significance was determined by one-way ANOVA followed by Bonferroni test

To investigate the biological significance of Adora2a expressed on vascular ECs or macrophages in retinal neovascularization, *Cdh5-Cre* and *Lysm-Cre* mice were bred with *Adora2a* ^flox/flox^ mice to generate *Adora2a* ^flox/flox^;*Cdh5-Cre* (*Adora2a* ^*VEC-KO*^), and *Adora2a* ^flox/flox^; *Lysm-Cre* (*Adora2a* ^*Mφ-KO*^) mice, respectively. The littermate *Adora2a* ^flox/flox^ mice (*Adora2a* ^*WT*^) mice were used as wild-type (WT) controls. After mice were subjected to the OIR, we performed isolectin B4 analysis of whole-mount retinas and observed that both EC- and macrophage-specific *Adora2a* deletion reduced the avascular area compared with the avascular area in *Adora2a* ^*WT*^ mice at P17 in the OIR model (Fig. 2a). However, with the loss of endothelial, but not macrophage *Adora2a*, we observed significantly less pathological neovascularization compared with WT controls (7.85 ± 0.33% vs. 3.31 ± 0.39% of total retina area; *P* < 0.001 using one-way ANOVA followed by Bonferroni test; Fig. 2a). To assess whether increasing the ischemic drive could reveal a role for macrophage *Adora2a* on retinal neovascularization, we exposed mice to 75% (rather than 70%) O_2_; this resulted in a larger area of avascular retina, but failed to demonstrate a decrease in pathological neovascularization with loss of macrophage-specific *Adora2a* compared to WT controls (Fig. 2b). Collectively, these results suggest that genetic *Adora2a* deletion from ECs but not macrophages suppresses pathological neovascularization during OIR. We also investigated the effect of endothelial-specific *Adora2a* deletion on vascular regression at P12 and observed that there was no significant difference in retinal vaso-obliteration following the hyperoxic phase between the *Adora2a* ^*VEC-KO*^and *Adora2a* ^*WT*^ groups (Supplementary Fig. 3), indicating that the reduced avascular area in *Adora2a* ^*VEC-KO*^ mice at P17 is ascribed to the modulation of revascularization at the hypoxic phase, rather than the alteration of vaso-obliteration at the hyperoxic phase.

To study whether endothelial-specific deletion of *Adora2a* affected developmental angiogenesis in the retina, we analyzed the development of the retinal vascular networks at P5, P7, P12, and P17 of *Adora2a* ^*VEC-KO*^ and control mice under RA by isolectin B4 staining of whole-mounted retinas. Quantitative analysis of the vascularized retinal area at P5 and P7, as well as the vascular density of three retinal vascular layers (the superficial, intermediate and deep layers) at P12 and P17, found no significant difference in the distribution and density of retinal vascularization between *Adora2a* ^*VEC-KO*^ and *Adora2a* ^*WT*^ mice (Supplementary Fig. 4).

To further evaluate hypoxia-induced pathologic angiogenesis in the retinas of these animals, we performed hematoxylin and eosin (H&E) and Ki-67 staining. Histologic examination revealed that OIR-induced infiltration of non-ganglion cells and neovascular nuclei from the inner limiting membrane into the vitreous were significantly reduced in *Adora2a* ^*VEC-KO*^ mice, compared with that of WT mice (Fig. 2c, d). Additionally, we observed a dramatic decrease of proliferative vascular ECs in *Adora2a* ^*VEC-KO*^mice, as assessed by double immunofluorescence staining of the proliferation marker Ki-67 and the endothelial nuclear marker ETS related gene (ERG)^20, 21^ (Fig. 2e, f). Taken together, these data suggest that endothelial *Adora2a* activation plays a causal role in pathological angiogenesis in the OIR model.

### Increased ADORA2A expression in hypoxic HRMECs via HIF-2α

**Fig. 3:**
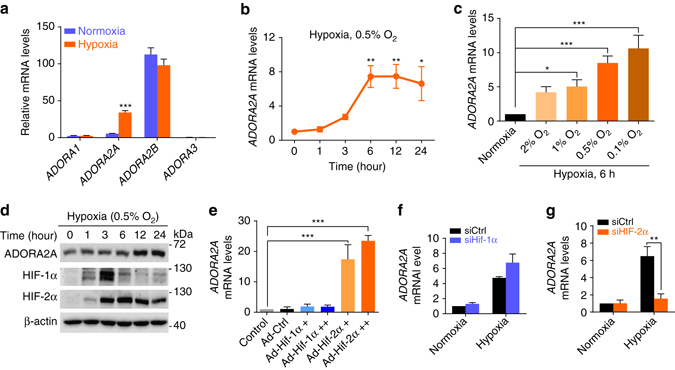
Hypoxia upregulates ADORA2A expression by activating HIF-2α in HRMECs. **a** Real-Time PCR analysis of mRNA expression for adenosine receptors in HRMECs. HRMECs were exposed to normoxia (21% O_2_) or hypoxia (0.5% O_2_) for 6 h. *n* = 4. ***P < 0.001 vs. normoxia. **b**, **c** Real-Time PCR analysis of *ADORA2A* mRNA expression in HRMECs. HRMECs were exposed to normoxia (21% O_2_) or hypoxia (0.5% O_2_) for indicated times **b** or with indicated O_2_ concentrations for 6 h **c**. *n* = 4. *P < 0.05; **P < 0.01, ***P < 0.001 vs. normoxia. **d** Western blot analysis of ADORA2A protein expression in HRMECs exposed to hypoxia (0.5% O_2_) for indicated times. *n* = 4. HIF-1α and HIF-2α were used as positive controls for hypoxia, and β-actin was used as loading control. **e** Real-Time PCR analysis of *ADORA2A* mRNA expression in HRMECs. HRMECs were infected with 10 ( + ) or 30 ( +  + ) pfu per cell of either Ad-mutHIF-1α, Ad-mutHIF-2α, or Ad-Ctrl. *n* = 3. *P < 0.001. **f**, **g** Real-Time PCR analysis of *ADORA2A* mRNA expression in HRMECs. HRMECs were transfected with siHIF-1α **f**, siHIF-2α **g**, or siCtrl. Forty-eight hours later, cells were exposed to hypoxia (0.5% O_2_) or air (21% O_2_) for an additional 12 h. *n* = 4. **P < 0.01. Data are represented as means ± s.e.m. Statistical significance was determined by unpaired Student’s *t*-test

Given the observation that endothelial Adora2a activation is critical for hypoxia-induced pathological angiogenesis during OIR and that Adora2a expression is increased in mouse OIR retinas, we next tested whether hypoxia upregulates ADORA2A expression in HRMECs. Strikingly, primary cultured HRMECs exposed to hypoxia exhibited a significant increase in mRNA expression of *ADORA2A*, but not *ADORA1*, *ADORA2B*, and *ADORA3* (Fig. 3a), and the extent of this increase closely correlates with the length of exposure time and the severity of hypoxia (Fig. 3b, c). Similar changes also occurred in the expression of ADORA2A at the protein level (Fig. 3d). HIFs, particularly HIF-1α and HIF-2α, mediate expression of numerous genes under hypoxic conditions. We investigated the role of these two HIFs in regulating ADORA2A in HRMECs. To this end, we first carried out HIF gain-of-function studies using adenoviral vectors encoding mutant HIF-1α (Ad-mutHIF-1α) or mutant HIF-2α (Ad-mutHIF-2α), which are both stable and transcriptionally active under normoxic conditions. Elevation of HIF mRNA levels in Ad-mutHIF-infected cells was confirmed by Real-Time PCR (Supplementary Fig. 5a, b). We found that HIF-2α, but not HIF-1α, upregulated *ADORA2A* mRNA in HRMECs (Fig. 3e). To confirm these findings, we also performed HIF loss-of-function studies in which we silenced the gene of HIF-1α or HIF-2α in HRMECs using short interfering RNA (siRNA) (Supplementary Fig. 5c, d). Knockdown of HIF-2α, but not HIF-1α, reversed ADORA2A upregulation in HRMECs exposed to hypoxia (Fig. 3f, g). These findings indicate that hypoxia induces ADORA2A expression via HIF-2α-dependent mechanisms in HRMECs.

### ADORA2A-regulated proliferation and sprouting of HRMECs

**Fig. 4:**
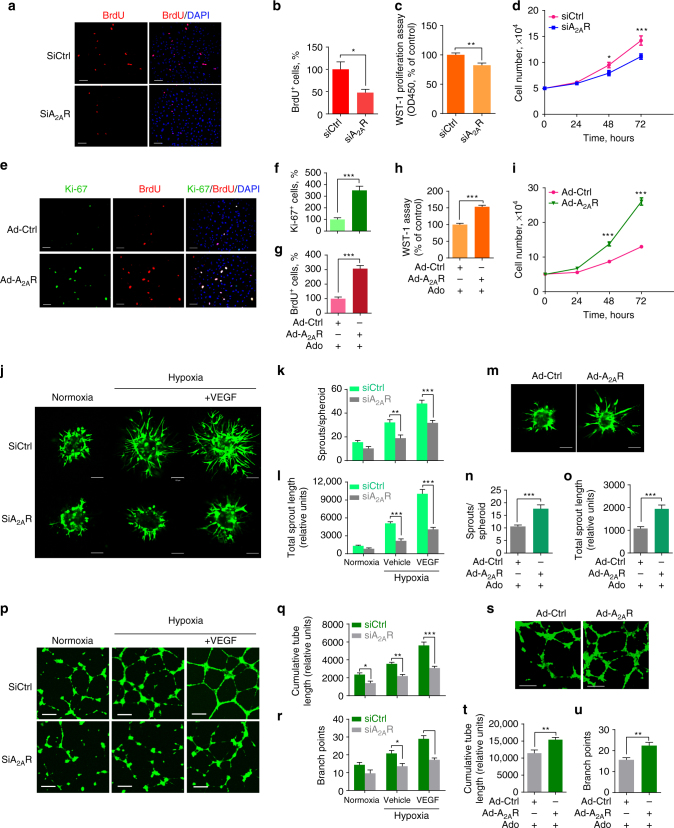
ADORA2A regulates HRMEC proliferation, sprouting and tube formation. **a**, **b** Bromodeoxyuridine (BrdU) staining of HRMECs transfected with siRNAs targeting human *ADORA2A* (siA_2A_R) or with a non-targeting negative control (siCtrl) under hypoxia conditions (0.5% O_2_). *n* = 6. *P < 0.05. **c** Cell proliferation measured by WST-1 cell proliferation assay. *n* = 6. **P < 0.01. **d** Growth curves of transfected cultures. *n* = 6. *P < 0.05, ***P < 0.001 vs. siCtrl. **e**–**g** Ki-67 and BrdU staining of HRMECs. HRMECs were infected with a recombinant adenovirus vector expressing human *ADORA2A* (Ad-A_2A_R) or a negative control adenovirus (Ad-Ctrl) in the presence of adenosine. *Scale bars*: 50 μm. *n* = 6. ***P < 0.001. **h** Cell proliferation measured by WST-1 cell proliferation assay. *n* = 6. ***P < 0.001. **i** Growth curves of HRMECs over 72 h following infection with Ad-Ctrl or Ad-A_2A_R in the presence of adenosine. *n* = 6. ***P < 0.001 vs. Ad-Ctrl. **j**–**u** HRMECs were transfected with siA_2A_R or siCtrl, or infected with Ad-Ctrl or Ad-A_2A_R, and then were cultured in collagen gel to grow into 3D multicellular spheroids, or on a 2D matrix to form a tube network in the presence or absence of VEGF or adenosine. **j**, **m** Representative *images* of spheroidal sprouting after culturing for 24 h in collagen matrix under hypoxia (0.5% O_2_) or normoxia (21% O_2_). *Scale bars*: 100 μm. Morphometric quantification of spheroid sprouting by calculating the number of sprouts per spheroid **k**, **n** as well as total sprout length **l**, **o**. *n* = 10 per group. *n* is number of spheroids quantified. **P < 0.01; ***P < 0.001. Representative *fluorescence photographs* of angiogenic tube formation **p**, **s**. *Scale bars*: 200 μm. Cumulative tube length quantified using the Image J software **q**, **t**, and branch points calculated from five experiments in each case **r**, **u**. *P < 0.05; **P < 0.01; ***P < 0.001. Data are represented as means ± s.e.m. Statistical significance was determined by unpaired Student’s *t*-test (for **b**, **c**, **f**, **g**, **h**, **k**, **l**, **n**, **o**, **q**, **r**, **t**, **u**) and two-way ANOVA followed by Bonferroni test (for **d**, **i**)

To evaluate the effect of ADORA2A on HRMEC proliferation, we performed BrdU incorporation, Ki-67 reactivity, and WST-1 proliferation assays, as well as cell number counting using HRMECs transfected with *ADORA2A* siRNA. Reduction of mRNA and protein levels of ADORA2A in silenced cells were confirmed by Real-Time PCR (Supplementary Fig. 6a) and western blot (Supplementary Fig. 6b), respectively. These assays consistently showed that *ADORA2A* knockdown markedly decreased HRMEC proliferation under hypoxia conditions (Fig. 4a–d). Similar results were obtained using the ADORA2A antagonist ZM241385 (Supplementary Fig. 7a, b). We found that *ADORA2A* knockdown also reduced EC proliferation under normoxic conditions (Supplementary Fig. 7c, d), though only modestly. Conversely, overexpression of ADORA2A by infection with adenovirus carrying the *ADORA2A* gene (Ad-A_2A_R) elevated ADORA2A protein levels (Supplementary Fig. 6c) and promoted HRMEC proliferation (Supplementary Fig. 7e–h). This proliferation of ADORA2A-overexpressing HRMECs was further enhanced by adding exogenous adenosine (Fig. 4e–i). In the three-dimensional spheroid sprouting assay, *ADORA2A* knockdown reduced hypoxia and VEGF-induced vessel sprouting (Fig. 4j–l). *ADORA2A* overexpression modestly increased sprout numbers and length under basal conditions (Supplementary Fig. 7i–k). This increased sprouting was further augmented in the presence of adenosine (Fig. 4m–o). These findings were confirmed in another angiogenesis assay, where HRMECs form a 2D vessel network (Fig. 4p–u). Collectively, these data indicate a significant pro-angiogenic effect of ADORA2A activation in HRMECs.

### ADORA2A-associated glycolysis in retinal ECs

**Fig. 5:**
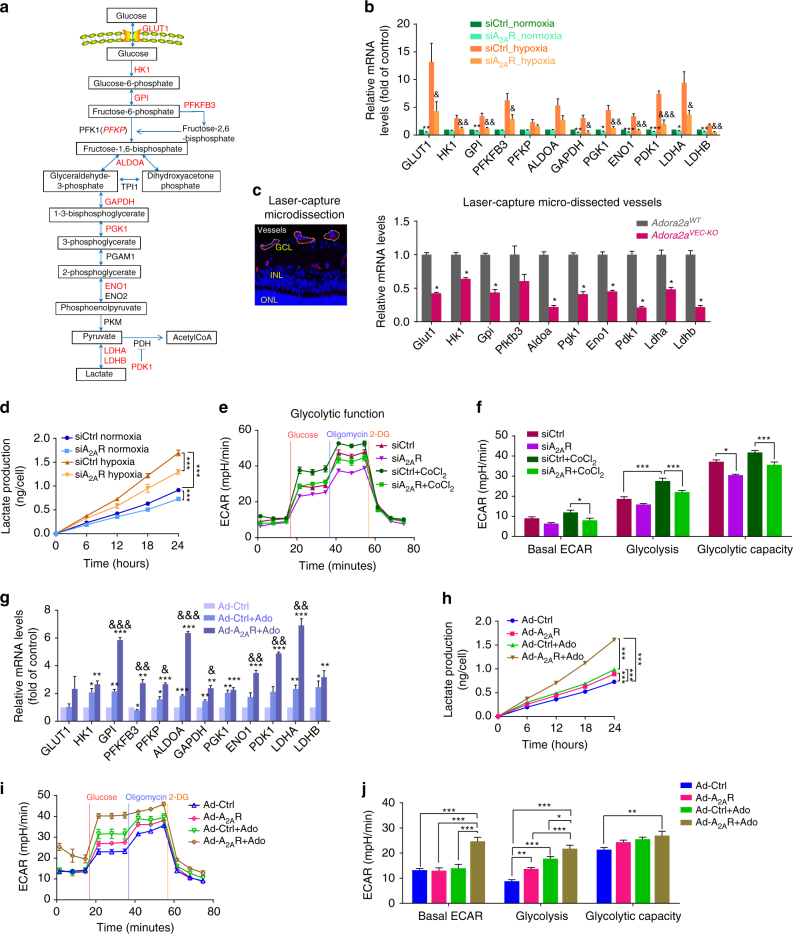
Adenosine-ADORA2A signaling regulates glycolysis in HRMECs and mouse retinal ECs. **a** Scheme showing the glycolytic pathway and associated enzymes. **b** Real-Time PCR analysis of the mRNA levels of glycolytic genes in HRMECs transfected with siA_2A_R or siCtrl under normoxia (21% O_2_) or hypoxia (0.5% O_2_). *n* = 5. *P < 0.05, **P < 0.01, ***P < 0.001 vs. siCtrl normoxia group; ^&^ *P* < 0.05, ^&&^ *P* < 0.01, ^&&&^ *P* < 0.001 vs. siCtrl hypoxia group. **c** Real-Time PCR analysis of the mRNA levels of glycolytic genes in retinal blood vessels isolated with laser-capture microdissection from OIR-*Adora2a* ^*WT*^ and *Adora2a* ^*VEC-KO*^ mice at P17. *n* = 4. *P < 0.05 vs. *Adora2a* ^*WT*^ group. **d** Levels of secreted lactate of HRMECs transfected with siA_2A_R or siCtrl under normoxia or hypoxia for indicated times. *n* = 3. ***P < 0.001. **e** ECAR profile showing glycolytic function in siCtrl- and siA_2A_R-transfected cells under hypoxia (0.5% O_2_) or normoxia (21% O_2_). *Vertical lines* indicate the time of addition of glucose (10 mmol/l), oligomycin (2 μmol/l), and 2-DG (50 mmol/l). **f** Quantification of glycolytic function parameters from **e**. *n* = 8 for normoxic groups and *n* = 16 for each of CoCl_2_ treatment groups. *P < 0.05; ***P < 0.001. **g** Real-Time PCR analysis of the mRNA levels of glycolytic genes in HRMECs infected with Ad-Ctrl or Ad-A_2A_R, with or without adenosine treatment. *n* = 4. *P < 0.05; **P < 0.01; ***P < 0.001 vs. Ad-Ctrl; ^&^ *P* < 0.05; ^&&^ *P < *0.01; ^&&&^ *P* < 0.001 vs. Ad-Ctrl + Ado. **h** Levels of secreted lactate of HRMECs infected with Ad-A_2A_R or Ad-Ctrl with or without adenosine treatment. *n* = 3. ***P < 0.001 vs. Ad-Ctrl. **i** ECAR profile showing glycolytic function in Ad-Ctrl- and Ad-A_2A_R-infected cells, with or without adenosine treatment. **j** Quantification of glycolytic function parameters from **i**. *n* = 8 per group. *P < 0.05; **P < 0.01; ***P < 0.001 vs. Ad-Ctrl. Data are represented as means ± s.e.m. Statistical significance was determined by unpaired Student’s *t*-test (for **b**, **c**, **g**), one-way ANOVA followed by Bonferroni test (for **f**, **j**), and two-way ANOVA followed by Bonferroni test (for **d**, **h**)

EC glycolysis plays a critical role in vessel sprouting and angiogenesis^22^. In response to hypoxia, HRMECs upregulated mRNA levels of key glycolytic enzymes, including GLUT1, HK1, GPI, PFKFB3, PFK1, ALDOA, GAPDH, PGK1, ENO1, PDK1, LDHA, and LDHB (Fig. 5a, b). The mRNA levels of these glycolytic genes were moderately reduced in *ADORA2A* knockdown HRMECs under normoxia compared with those of control HRMECs. Under hypoxia conditions, *ADORA2A* knockdown remarkably downregulated mRNA levels of these glycolytic genes. Protein levels of PFKFB3, a key glycolytic activator^8, 9^, are also reduced in hypoxic HRMECs treated with siRNAs targeting human *ADORA2A* (siA_2A_R, Supplementary Fig. 8) and OIR retinas from *Adora2a* ^*VEC-KO*^mice (Supplementary Fig. 9).To test whether similar changes also take place in retinal vessels or ECs in vivo, we used laser-capture microdissection to isolate retinal vessels and anti-CD31 antibody-conjugated magnetic beads to isolate mouse retinal ECs from *Adora2a* ^*VEC-KO*^ and control mice, respectively, and analyze gene expression specifically in vessels or ECs by Real-Time PCR. Consistent with the findings from the in vitro data, most of the key glycolytic genes were significantly downregulated in vessels (Fig. 5c) or ECs (Supplementary Fig. 10) isolated from the OIR retinas of *Adora2a* ^*VEC-KO*^ mice compared with controls.

Lactate is the metabolite generated in the glycolytic pathway by lactate dehydrogenase A (LDHA) and functions as a signaling molecule for angiogenesis^10, 22^. Hypoxia resulted in a robust increase in the level of secreted lactate by HRMECs, but this increase was attenuated by transfection of *ADORA2A* siRNA into HRMECs (Fig. 5d). Using Seahorse Flux analysis, we further assessed the glycolytic function of HRMECs directly by measuring the extracellular acidification rate (ECAR). As shown in Fig. 5e, f, *ADORA2A* knockdown in HRMECs significantly reduced glucose-induced glycolysis and also reduced maximal glycolytic capacity. Basal cellular oxygen consumption (OCR), indicative of mitochondrial respiratory activity, and ATP production were not affected by *ADORA2A* deletion (Supplementary Fig. 11a, b) or overexpression (Supplementary Fig. 11c, d), whereas maximal respiration and spare respiratory capacity were decreased in Ad-A_2A_R plus adenosine-treated cells. Altogether, these results demonstrate that ADORA2A is an endogenous regulator of glycolysis in retinal ECs.

We next explored whether ADORA2A activation *per se* without hypoxia is able to increase EC glycolysis. *ADORA2A* overexpression or adenosine treatment alone resulted in a modest increase in expression of key glycolytic genes, lactate production and ECAR in HRMECs (Fig. 5g–j). Interestingly, these increased parameters were strikingly further elevated when adenosine was added to *ADORA2A*-overexpressing HRMECs compared with those from control cells or cells treated with adenosine alone (Fig. 5g–j), supporting that both ADORA2A upregulation and adenosine availability are crucial for HRMEC glycolysis.

To further confirm the findings obtained through genetic approaches, we also used the ADORA2A antagonist ZM241385 and ADORA2A agonist CGS21680 to block or activate ADORA2A in HRMECs, respectively. As shown in Supplementary Fig. 12, the levels of secreted lactate were decreased in ZM241385-treated cells and increased in CGS21680-treated cells compared with vehicle treated controls under hypoxic conditions.

### ADORA2A-mediated glycolysis in HRMEC angiogenesis in vitro

**Fig. 6:**
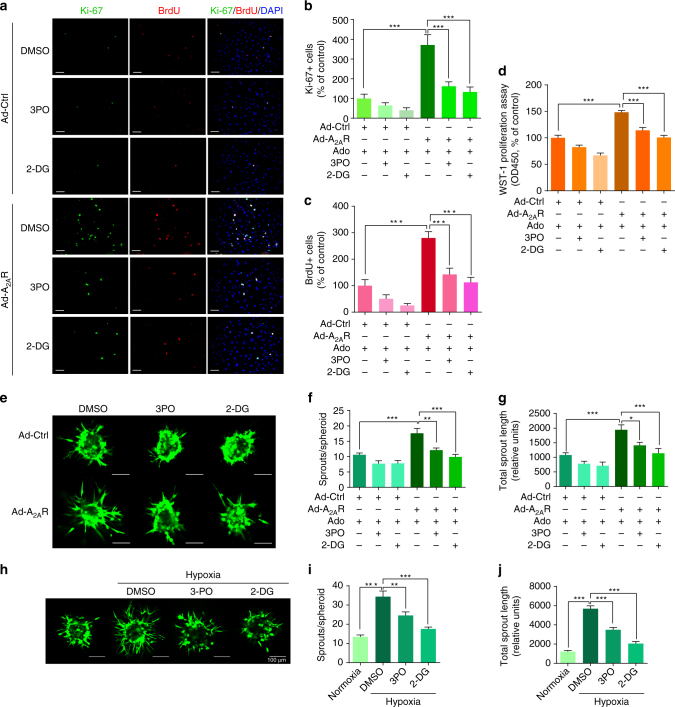
Glycolysis is involved in ADORA2A activation-mediated HRMEC proliferation and sprouting. **a**–**g** Results of Ki-67 and BrdU staining **a**–**c**, WST-1 cell proliferation assay **d** and EC spheroid sprouting assay **e**–**g** in HRMECs. HRMECs were infected with Ad-Ctrl or Ad-A_2A_R adenoviruses, and then treated with adenosine in the presence or absence of two different glycolytic inhibitors (3PO, 10 μM, and 2-DG, 5 mM). *n* = 6. *P < 0.05; **P < 0.01; ***P < 0.001. *Scale bars*: 50 μm for **a**; *Scale bars*: 100 μm for **e**. **h**–**j** HRMECs were cultured as 3D multicellular spheroids in the presence or absence of 3PO or 2DG. Representative images of spheroidal sprouting after culturing for 24 h in collagen matrix under hypoxia (0.5% O_2_) or normoxia (21% O_2_) **h**. Morphometric quantification of spheroid sprouting by calculating the number of sprouts per spheroid **i** and total sprout length **j**. *n* = 10 per group (*n* is number of spheroids quantified). *P < 0.05, **P < 0.01; ***P < 0.001. *Scale bars*: 100 μm. Data are represented as means ± s.e.m. Statistical significance was determined by one-way ANOVA followed by Bonferroni test

After having shown that ADORA2A plays a crucial role in HRMEC glycolysis, we examined whether inhibition of glycolysis could block the ADORA2A-mediated angiogenic effects. As expected, *ADORA2A* overexpression induced HRMEC proliferation and subsequent sprouting in the presence of adenosine (Fig. 6a–g). Blockade of glycolysis by the PFKFB3 inhibitor 3-(3-pyridinyl)-1-(4-pyridinyl)-2-propen-1-one (3PO) or the non-metabolizable glucose analog 2-deoxy-d-glucose (2-DG) significantly reversed the ADORA2A-mediated pro-angiogenic effect. We further examined if glycolysis blockade is able to inhibit HRMEC spheroid sprouting when ADORA2A is activated by hypoxia. Indeed, 3PO or 2-DG treatment dramatically decreased hypoxia-mediated hypersprouting (Fig. 6h–j). Overall, these data suggest that ADORA2A-mediated glycolysis has a critical role in the ADORA2A activation-driven angiogenic response.

### ADORA2A and glycolysis in the formation of endothelial tips

**Fig. 7:**
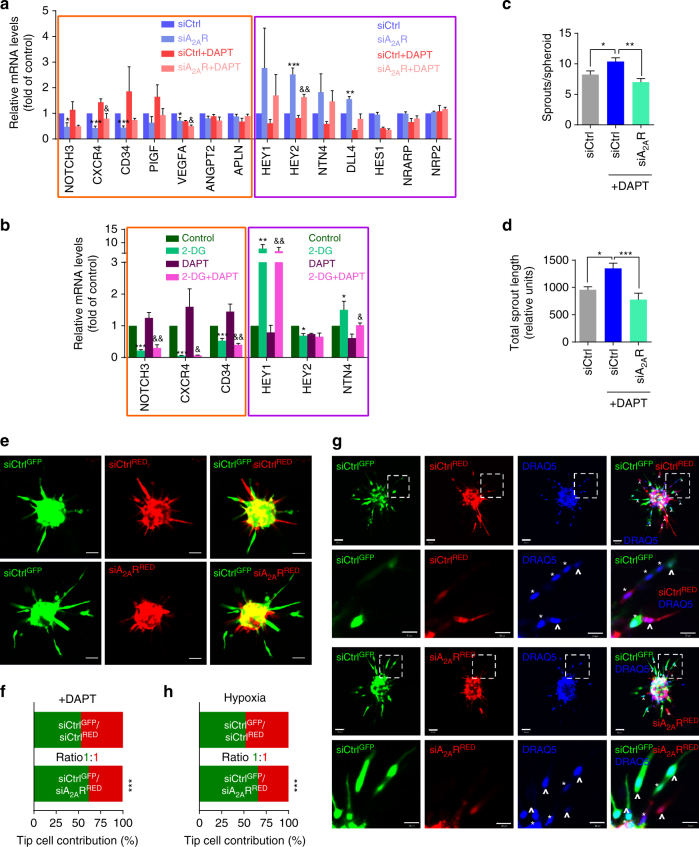
ADORA2A regulates HRMEC tip cell formation. **a** Real-Time PCR analysis of the mRNA levels of the sprouting-governing genes in HRMECs transfected with siA_2A_R or siCtrl, in the presence or absence of DAPT (*n* = 3; *P < 0.05; **P < 0.01; ***P < 0.001 vs. siCtrl; ^&^ *P* < 0.05, ^&&^ *P* < 0.01 vs. siCtrl + DAPT). **b** Real-Time PCR analysis of the mRNA levels of the sprouting-governing genes in 2-DG-treated HRMECs in the presence or absence of DAPT (*n* = 4; *P < 0.05; **P < 0.01; ***P < 0.001 vs. vehicle control; ^&^ *P* < 0.05, ^&&^ *P* < 0.01 vs. DAPT). **c**, **d** Morphometric quantification of spheroid sprouting from cells transfected with siCtrl or siA_2A_R in the presence or absence of DAPT. *n* = 10 per group. *n* is number of spheroids quantified. *P < 0.05; **P < 0.01; ***P < 0.001. **e** Representative fluorescence photographs of EC spheroids containing a 1:1 mixture of siCtrl^GFP^ and siCrl^RED^ ECs, or a 1:1 mixture of siCtrl^GFP^ ECs and ECs with siA_2A_R^RED^ in the presence of DAPT. *Scale bars*, 100 μm. **f** Quantification of the fraction of tip cells with the indicated genotypes shown in **e** (*n* = 10; ***P < 0.001 vs. siCtrl^GFP^). **g** Representative fluorescence photographs of mosaic EC spheroids containing a 1:1 mixture of siCtrl^GFP^ ECs and siA_2A_R^RED^ ECs under hypoxia conditions. Cells were stained with DRAQ5 (*blue*) to mark EC nuclei. Tip cells are indicated by “^“; Stalk cells are indicated by “*“. The *2nd* and *4th* rows are magnification of the *boxed* regions in the *1st* and *3rd rows*, respectively. *Scale bar*: 50 μm (*1st* and *3rd* rows) and 20 μm (*2nd* and *4th* rows). **h** Quantification of the fraction of tip cells with the indicated genotypes shown in **g** (*n* = 10 per group). ***P < 0.001 vs. siCtrl^GFP^. Data are represented as means ± s.e.m. Statistical significance was determined by unpaired Student’s *t*-test (for **a**, **b**, **f**, **h**) and one-way ANOVA followed by Bonferroni test (for **c**, **d**)

Vascular growth is led by endothelial tip cells and supported by proliferative stalk cells, and each of these cell types is signatured with an array of unique genes^23^. To examine whether ADORA2A and glycolysis modulate the formation of endothelial tip cells, we measured the expression of these signature genes. In HRMECs, *ADORA2A* knockdown downregulated the tip-cell-enriched genes (*CXCR4, CD34, and VEGFA*), and upregulated the stalk-cell-enriched genes (*HEY1, HEY2, NTN4, and DLL4*) under baseline conditions (Fig. 7a). Blocking of Notch signaling using the γ-secretase inhibitor DAPT (*N*-[*N*-(3,5-difluorophenacetyl)-l-alanyl]-S-phenylglycine t-butylester) promoted a tip cell phenotype^24^. Indeed, DAPT treatment caused an expected increase in the expression of tip-cell-enriched genes and downregulation of stalk-cell-specialized genes, whereas *ADORA2A* knockdown (Fig. 7a) or 2-DG-mediated glycolysis blockade (Fig. 7b) counteracted the DAPT-mediated alteration of the genetic tip vs. stalk cell signature upon Notch signaling blockade. Accordingly, pharmacological inhibition of Notch signaling in HRMECs has been shown to increase the number and length of sprouts, but this hyper-sprouting was abolished when cells were treated with *ADORA2A* siRNA (siA_2A_R, Fig. 7c, d). Using a model of mosaic spheroids, we further assessed whether *ADORA2A* knockdown would influence tip cell formation induced by Notch signaling inhibition. HRMECs were infected with adenovirus encoding mCherry (red fluorescence) or EGFP (green fluorescence), then transfected with either siCtrl (control) or siA_2A_R. The consequent siCtrl^GFP^, siCtrl^RED^_,_ or siA_2A_R^RED^ cells were mixed in a 1:1 ratio to generate mosaic spheroids. In control spheroids, generated by mixing siCtrl^RED^ and siCtrl^GFP^ cells, a comparable fraction of siCtrl^GFP^ and siCtrl^RED^ ECs were observed at the tip position (Fig. 7e, f). In contrast, in spheroids containing a 1:1mixture of siA_2A_R^RED^ and siCtrl^GFP^ cells in the presence of DAPT, a reduced number of siA_2A_R^RED^ ECs was identified at the tip. Similar results were obtained with HRMECs under hypoxic conditions (Fig. 7g, h).

### ADORA2A activation induces HRMEC glycolysis via HIF-1α

**Fig. 8:**
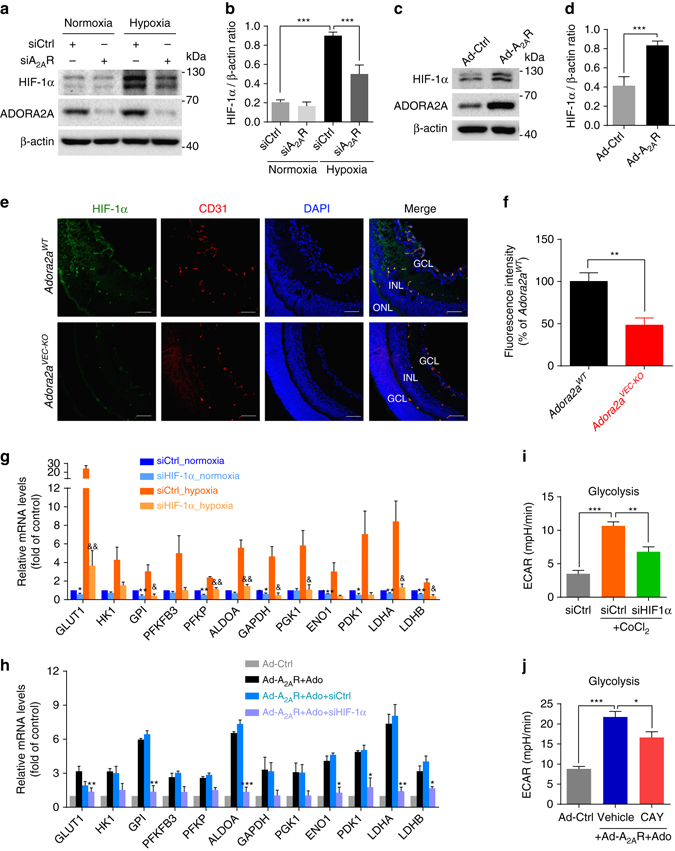
ADORA2A activation mediates increase in glycolysis via a HIF-1α-dependent pathway. **a**–**d** Western blot analysis of HIF-1α mRNA and protein expression in HRMECs transfected with siA_2A_R or siCtrl under hypoxia (0.5% O_2_) or normoxia (21% O_2_) **a**, **b**, or infected with Ad-Ctrl or Ad-A_2A_R under normoxia **c**, **d**. *n* = 4. ***P < 0.001. **e**, **f** HIF-1α immunofluorescent staining of OIR retinas from *Adora2a* ^*WT*^ and *Adora2a* ^*VEC-KO*^ mice at postnatal day (P)15. Representative *green* (HIF-1α), *red* (CD31), *blue* (nuclei, DAPI), and merged images captured with confocal fluorescent microscopy. *GCL* ganglion cell layer, *INL* inner nuclear layer, *ONL* outer nuclear layer. *Scale bar*, 50 μm. The fluorescence intensity of HIF-1α staining was calculated by Image J software then normalized to that of WT control (*Adora2a* ^*WT*^). *n* = 6 mice for each group. **P < 0.01. **g** Real-Time PCR analysis of the mRNA levels of glycolytic genes in HRMECs transfected with siHIF-1α or siCtrl under normoxia (21% O_2_) or hypoxia (0.5% O_2_). *n* = 3. *P < 0.05; **P < 0.01; ***P < 0.001 vs. siCtrl normoxia group; ^&^ *P* < 0.05; ^&&^ *P* < 0.01 vs. siCtrl hypoxia group. **h** Real-Time PCR analysis of the mRNA levels of glycolytic genes in HRMECs. Cells were transfected with HIF-1α siRNA or siCtrl for 24 h under normoxia, and further infected with Ad-A_2A_R or Ad-Ctrl for an additional 24 h, followed by adenosine treatment for another 12 h. *n* = 4. *P < 0.05; **P < 0.01; ***P < 0.001 vs. Ad-A_2A_R + Ado + siCtrl group. **i** Quantification of glycolytic function in HRMECs transfected with siCtrl or siHIF-1α in the presence of CoCl_2_ (200 μM). *n* = 8 per group. *P < 0.05; ***P < 0.001. **j** Quantification of glycolytic function in HRMECs infected with Ad-Ctrl or Ad-A_2A_R, with or without adenosine treatment, in the presence or absence of the HIF-1α inhibitor CAY10585. *n* = 7, 7, 6, respectively. *P < 0.05; ***P < 0.001. Data are represented as means ± s.e.m. Statistical significance was determined by unpaired Student’s *t*-test (for **d**, **f**, **g**, **h**) and one-way ANOVA followed by Bonferroni test (for **b**, **i**, **j**)

HIF-1 is the principal regulator of the transcriptional response to hypoxia. Almost all enzymes of the glycolytic cascade, as well as glucose transport proteins, are upregulated by HIF-1α^25, 26^. Therefore, we hypothesized that HIF-1α might mediate the regulation effect of ADORA2A on EC glycolysis. To test this, we first analyzed the effects of *ADORA2A* knockdown or overexpression on HIF-1α expression in HRMECs. We found that HIF-1α mRNA levels in HRMECs were indistinguishable between control and *ADORA2A* knockdown or overexpression groups (Supplementary Fig. 13). In contrast, evaluation of HIF-1α protein expression in siA_2A_R or Ad-*A*_*2A*_ *R*- infected HRMECs by western blot showed reduced or increased HIF-1α protein levels, respectively (Fig. 8a–d). Consistent with these observations, EC-specific *ADORA2A* deletion decreased the expression of HIF-1α in retinal ECs of OIR mice at P15 (initial stages of neovessel formation) (Fig. 8e, f). Furthermore, we examined the role of HIF-1α in ADORA2A activation-mediated upregulation of glycolytic genes in HRMECs. Both hypoxia and Ad-*A*_*2A*_ *R*/adenosine-driven ADORA2A activation upregulated the key glycolytic enzymes (Fig. 8g, h) and enhanced glycolytic function (Fig. 8i, j) in HRMECs. These effects were abolished by siHIF-1α or treatment with the specific HIF inhibitor CAY10585. Altogether, these data demonstrate that activation of ADORA2A promotes glycolysis in HRMECs by enhancing HIF-1α protein accumulation without altering its mRNA level.

### ADORA2A regulates HIF-1α level via translational pathways

**Fig. 9:**
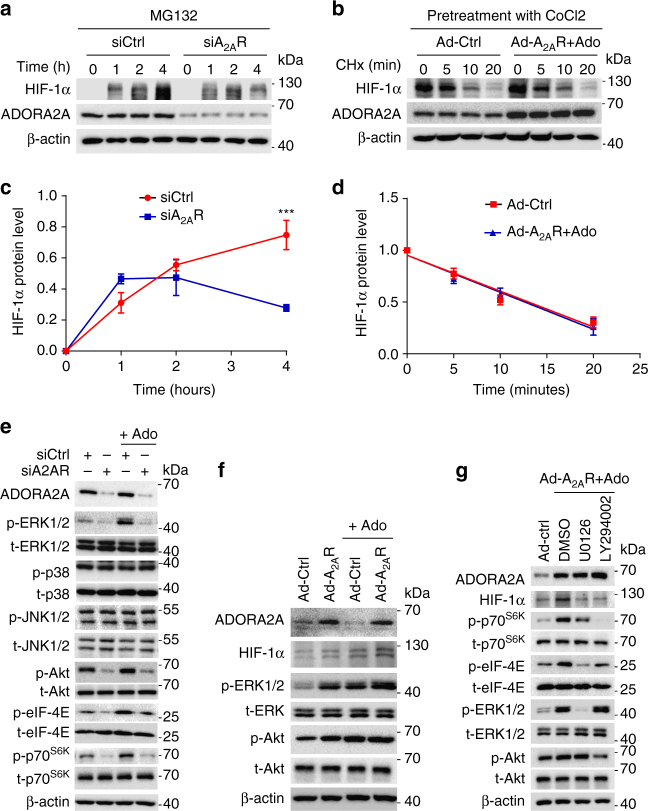
ADORA2A regulates HIF-1α protein level through activation of the PI3K/Akt and MEK/ERK-mediated translational machinery. **a** Western blot analysis of HIF-1α at protein level in HRMECs. Cells were transfected with siA_2A_R or the siCtrl for 48 h, and then treated with MG-132 (10 µM) for 1, 2 and 4 h. **b** Western blot analysis of HIF-1α at protein level in HRMECs. Ad-Ctrl and Ad-A_2A_R-infected HRMECs were first treated with CoCl_2_ (200 μM) for 8 h to increase HIF-1α protein level and then further treated with CHx (50 µM) for the indicated time periods. **c**, **d** The relative protein levels of HIF-1α were quantified by comparing the intensities of protein bands at the indicated times to that at time 0. *n* = 3, ***P < 0.001. Data are represented as means ± s.e.m. Statistical significance was determined by two-way ANOVA followed by Bonferroni test. **e** Western blot analysis of the levels of total-ERK (t-ERK), phospho-ERK1/2 (p-ERK1/2), phospho-p38 (p-p38), total-p38 (t-p38), phospho-JNK1/2 (p-JNK1/2), total-JNK1/2 (t-JNK1/2), phospho-p70^S6K^ (p-p70^S6K^), total-p70^S6K^ (t-p70^S6K^), phospho-eIF-4E (p-eIF-4E), and total-eIF-4E (t-eIF-4E) in HRMECs transfected with siA_2A_R or siCtrl, with or without adenosine treatment under hypoxia. *n* = 3. **f** Western blot analysis of HIF-1α and phosphoproteins in HRMECs infected with Ad-A_2A_R, with or without adenosine treatment. *n* = 3. **g** Western blot analysis of HIF-1α and phosphoproteins in HRMECs infected with Ad-Ctrl and Ad-A_2A_R in the presence of U0126 (10 μM) or LY294002 (10 μM). *n* = 4

In view of our findings that ADORA2A activation upregulates the protein level of HIF-1α without affecting transcriptional induction of HIF-1α, we next explored whether ADORA2A regulates HIF-1α translation or protein stability. To assess the effect of ADORA2A on HIF-1α mRNA translation, the proteasome inhibitor MG132 was used to avoid the degradation of HIF-1α protein. *ADORA2A* knockdown resulted in a much slower rate of MG132-induced HIF-1α accumulation (Fig. 9a, c), indicating that HIF-1α protein synthesis in HRMECs is markedly impaired by *ADORA2A* knockdown. To test the effect of ADORA2A on HIF-1α protein stability, the protein translation inhibitor cycloheximide (CHx) was employed to prevent de novo HIF-1α protein synthesis. In the presence of CHx, HIF-1α levels rapidly declined in HRMECs under hypoxia and ADORA2A overexpression did not modify the degradation rate of HIF-1α (Fig. 9b, d). Thus, ADORA2A modulates the protein synthesis of HIF-1α but not its degradation.

To characterize the underlying mechanisms by which ADORA2A regulates HIF-1α protein synthesis, we first surveyed several MAPK and PI3K/Akt signaling pathways. As shown in Fig. 9e, *ADORA2A* knockdown or adenosine treatment did not affect p38 kinase or JNK phosphorylation. However, we observed a profound activation of the ERK1/2 and Akt signaling pathways in HREMCs after adenosine treatment under hypoxia, and this activation was markedly reduced by *ADORA2A* knockdown. Furthermore, gain-of-function studies revealed more robust ERK1/2 and Akt phosphorylation after adenosine treatment in Ad-*ADORA2A*-infected HREMCs (Fig. 9f). These observations prompted us to hypothesize that ERK1/2 and Akt activation may govern the effects of ADORA2A activation on the enhancement of HIF-1α protein level. In fact, the MEK/ERK and PI3K/Akt signaling pathways have been demonstrated to be involved in HIF-1α translation via functional activation of the translation initiation factors p70^S6K^ and eIF-4E in various cells^27–29^. Indeed, in parallel with the alteration of ERK1/2 and Akt phosphorylation, *ADORA2A* knockdown significantly inhibited basal and adenosine-mediated activation of p70^S6K^ and eIF-4E (Fig. 9e). In addition, treatment of HRMECs with adenosine and Ad-A_2A_R effectively increased the protein level of HIF-1α (Fig. 9f), as well as the phosphorylation of both p70^S6K^ and eIF-4E, which was inhibited by the MEK inhibitor U0126 or PI3K inhibitor LY294002 (Fig. 9g). Collectively, these results suggest that ADORA2A regulates HIF-1α protein synthesis through the MEK/ERK and PI3K/Akt pathways that activate the HIF-1α translational machinery.

## Discussion

**Fig. 10:**
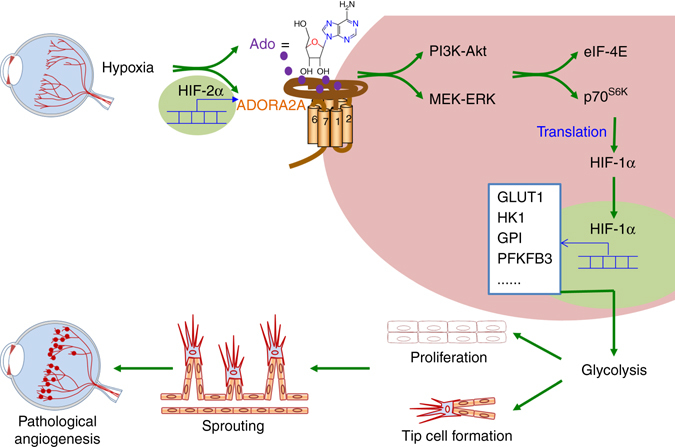
Schematic diagram illustrating the molecular mechanisms underlying the angiogenic effect of adenosine-ADORA2A-mediated signaling cascade

In this study, we demonstrate a novel cellular and molecular mechanism whereby endothelial ADORA2A activation promotes pathological angiogenesis in the retina. Retinal hypoxia leads to the activation of HIF-2α, which enhances ADORA2A expression. Increased ADORA2A in retinal ECs, in turn, enhances the accumulation of HIF-1α via a translational pathway. Elevated HIF-1α is largely responsible for elevation of glycolytic enzymes as well as endothelial glycolysis. ADORA2A-mediated glycolysis critically contributes to retinal EC proliferation, sprouting and angiogenesis (Fig. 10). These findings provide new insights into a previously unrecognized effect of ADORA2A on endothelial glycolysis in ischemic retinopathies and highlight the translational potential of targeting ADORA2A in the treatment of vision-threatening eye diseases.

Extracellular adenosine accumulates during hypoxia conditions and signals through four G-protein-coupled adenosine receptors (ADORA1, ADORA2A, ADORA2B, and ADORA3)^12^. Adenosine signaling events play a key role in various ischemic diseases, including ischemic proliferative retinopathies. In a previous study, *Adora2a* global knockout mice exhibited markedly suppressed retinal angiogenesis in the OIR model, which was accompanied by a low level of VEGF mRNA in the retina^30^. The critical role of VEGF in retinal angiogenesis has been long established by a large body of experimental studies and clinical application^31, 32^. EC-autonomous VEGF is indispensable for vascular homeostasis while VEGF produced by non-ECs is critical for the angiogenic cascade^33^. Therefore, the low level of VEGF mRNA in *Adora2a*-deficient retinas may be likely due to *Adora2a* deficiency in non-ECs, and the *Adora2a* deficiency in non-ECs may mainly account for the suppressed retinal angiogenesis in *Adora2a*-deficient mice. Adora2a in macrophages has been reported to be highly involved in VEGF production^34^. We suspected that *Adora2a* deficiency in macrophages may be the major reason for the low level of VEGF and decreased neovascularization in the retina. However, *Adora2a* deficiency in myeloid cells, although decreasing the avascular area, did not significantly suppress neovascularization in OIR retinas. In contrast, endothelial *Adora2a* deficiency dramatically decreased both avascular and neovascularization areas in retinas of OIR models, suggesting that endothelial *Adora2a* is critical for pathological retinal angiogenesis. Since endothelial autocrine VEGF signaling is dispensable in angiogenesis in vivo^33^, decreased VEGF production from *Adora2a*-deficient retinal ECs may explain only in part the decreased pathological angiogenesis in retinas of the OIR model in endothelial *Adora2a*-deficient mice.

Tumor cells mainly rely on aerobic glycolysis, a phenomenon termed “the Warburg effect,” to generate ATP and provide substances for fast growth^35^. Interestingly, vascular ECs use the same pathway to generate their energy. In ECs, glycolysis generates 85% of the total ATP^8^. Of this, ECs use 60% for maintenance of homeostasis and 40% for proliferation^36, 37^. PFKFB3 knockdown markedly suppressed angiogenesis, demonstrating the importance of glycolysis for endothelial angiogenesis^8–10^. Growth factors such as VEGF and FGF2 enhance the expression of the key glycolytic enzyme PFKFB3 and increase glycolysis to support the high ATP demands for vessel sprouting^8, 10^, indicating that glycolysis is also critical for growth factor-driven angiogenesis. Interestingly, we show here that knockdown or deletion of endothelial ADORA2A dramatically inhibited gene expression of most glycolytic enzymes, including PFKFB3, in vitro and in vivo, resulting in decreased glycolysis under hypoxia conditions. However, it seems that ADORA2A knockdown or blockade has less effect on EC glycolysis under normoxia conditions. This may be due to the low levels of ADORA2A and adenosine in normoxic cells. In contrast, *ADORA2A* overexpression significantly upregulated glycolytic enzymes and increased glycolysis in the presence of adenosine. Blocking glycolysis with 3PO and 2-DG dramatically inhibited proliferation and hyper-sprouting of *ADORA2A*-overexpressing ECs. Overall, these loss- and gain-of-function assays strongly support that ADORA2A-regulated glycolysis is one of the critical pathways for ADORA2A-mediated angiogenesis.

HIFs are currently viewed as the major regulators of oxygen homeostasis, angiogenesis, and vascular permeability via induction of a host of pro-angiogenic genes and glycolytic enzymes^38^. Proliferative retinopathies are characterized by hypoxia-induced pathological neovascularization driven by the HIF-1α and HIF-2α pathways^39–41^. HIF-2α is a master regulator of proangiogenic factors in retinal vascular ECs. HIF-2α haploinsufficiency results in a reduced ability to induce multiple proangiogenic factors and reduced neovascularization in the retinas of mice with OIR^41^. This study indicates HIF-2α is able to upregulate ADORA2A. Likely, the angiogenic effects of HIF-2α are, at least in part, mediated through ADORA2A-dependent signaling. Most of the glycolytic genes are identified as HIF-1α target genes^25, 26, 42^. The expression of HIF-1α can be modulated by different regulators through various mechanisms, including the basal transcription and translation machinery, as well as posttranscriptional and posttranslational mechanisms^43^. Ouyang et al.^44^ showed that ADORA2A activation rapidly induced HIF-1α mRNA expression via the cAMP/PKA/CREB pathway in macrophages. In contrast, *ADORA2A* knockdown or overexpression in retinal ECs did regulate the protein level of HIF-1α, but did not alter its mRNA level, implying that ADORA2A did not affect HIF-1α transcription or mRNA stability. Our study further revealed that ADORA2A preferentially activates PI3K/Akt and MEK/ERK, two critical pathways responsible for HIF-1α protein synthesis^27, 28, 45^, as well as the downstream translational regulatory proteins of p70^S6K^ and eIF-4E, without altering the phosphorylation of JNK1/2 and p38 MAPK kinase. Importantly, inhibitors of PI3K and MEK effectively blocked the ADORA2A activation-induced increase in HIF-1α protein, suggestive of a role for the PI3K and MEK-dependent translational machinery in ADORA2A-mediated upregulation of HIF-1α protein levels. Additionally, the adenylyl cyclase inhibitor SQ22536 and PKA inhibitor H-89 did not affect translational induction of HIF-1α (Supplementary Fig. 14), suggesting that the cAMP/PKA/CREB pathway is not involved in ADORA2A-mediated HIF-1α protein synthesis in HRMECs.

A salient observation of the present study is that *ADORA2A* knockdown impaired tip cell activity. Tip cells play a critical role in various models of sprouting angiogenesis, including proliferative retinopathy^46^. Tip cells are located at the forefront of vessel branches, and are highly polarized with numerous filopodia to probe the environment, and migrate toward angiogenic stimuli^47, 48^. Recently a critical role of glycolysis in tip cell formation has been demonstrated^8, 9^. Thus, a decrease in tip cell formation and tip cell activity for *ADORA2A* knockdown retinal ECs is, at least in part, dependent on ADORA2A-mediated glycolysis. Indeed, blockade of glycolysis by the glycolytic inhibitor 3PO and 2-DG markedly suppresses *ADORA2A* overexpression-induced endothelial sprouting. It has been shown that partial and transient reduction of glycolysis by PFKFB3 knockdown or blockade was not able to significantly alter the genetic tip or stalk cell signature of ECs^8, 9^. Similarly, we found that short term treatment with the ADORA2A agonist CGS12680 or ATL313 did not affect transcript levels of Notch1, DLL4 or Notch1 target genes HES1, HEY1, and HEY2 (Supplementary Fig. 15), indicating that ADORA2A activation probably does not directly regulate endothelial Notch signaling. However, we demonstrate here that systemic downregulation of glycolytic enzymes by *ADORA2A* knockdown or near-complete inhibition of glycolysis by 2-DG treatment downregulated tip-cell-enriched genes and upregulated stalk-cell-enriched genes. It is, therefore, likely that some of the glycolytic enzymes and/or their products regulate these genes. On the other hand, ADORA2A- or 2-DG-regulated molecules include genes that broadly impact glucose metabolism rather than selectively suppress glycolysis. For example, both *ADORA2A* knockdown and 2-DG treatment can inhibit hexokinase-1 (HK1), an enzyme that phosphorylates glucose to produce glucose-6-phosphate (G6P), the first step in glucose metabolism. As such, many other pathways associated with glucose metabolism including reactive oxygen species and the pentose phosphate pathway (PPP) are affected^7, 49^. This may contribute to the altered expression of tip-enriched genes. Additionally, it might be also possible that long-term or severe inhibition of glycolysis-associated signals by *ADORA2A* knockdown or 2-DG may eventually change endothelial homeostasis and consequently alter gene expression.

A recent genome-wide association study indicates a close association of vascular disease with the lead SNP in intronic regions of the *ADORA2A* gene^50^. In a recent study on PDR of patients with type 2 diabetes, microarray analysis of gene expression in fibrovascular membranes showed that the level of *ADORA2A* mRNA was much higher in samples excised from patients with PDR compared with those from patients with non-PDR^51^. In another PDR study, Charles et al. examined the associations between PDR and variants of the *ADORA2A* gene in a cohort of patients with type 1 diabetes. They found that among tagging SNPs (tSNPs; rs2236624-C/T, and rs4822489-G/T) in the *ADORA2A* gene, participants homozygous for the T allele displayed a decreased risk of developing prevalent PDR^19^. The data we collected from the GTEx database have shown that, compared with individuals homozygous for the C or G alleles, individuals homozygous for the T allele actually have a low level of *ADORA2A* mRNA in their blood cells (Supplementary Fig. 2), indicating that in Charles’s study, the observation that type 1 diabetic patients have a low risk of developing PDR is very likely due to a low level of *ADORA2A* expression in their retinal ECs. With the results from this study showing that endothelial *Adora2a* knockdown or deletion reduces pathological retina angiogenesis, it may be safe to conclude that highly expressed ADORA2A, especially in retinal ECs, contributes to the development of pathological angiogenesis in the human retina.

Inhibiting pathological angiogenesis by targeting angiogenic factors such as VEGF has become an attractive clinical strategy in the treatment of proliferative retinopathies^52^. However, this approach has only achieved limited success. Recent advances in the suppression of retinal angiogenesis in preclinical studies have included therapies targeting EC glycolysis. Blocking the glycolytic regulator PFKFB3 suppresses retinal neovascularization in mouse models of OIR and AMD^9, 10^. As shown in this study, ADORA2A is an upstream regulator for the HIF-1α-associated glycolytic pathway in retinal ECs, suggesting its dominant role in regulation of endothelial glycolysis and the associated angiogenic effect. Additionally, ADORA2A inhibition is also able to suppress VEGF-mediated angiogenesis. Hypoxia-mediated paracrine VEGF can induce EC expression of GLUT1^53^ and PFKFB3^8^ and subsequently promote glycolysis for vessel sprouting during ischemic retinopathies. Thus, the blocking of glycolysis by ADORA2A inactivation may also reduce the effect of VEGF on angiogenesis. Indeed, we found that *ADORA2A* knockdown led to a marked reduction in VEGF-induced HRMEC sprouting and tube formation, suggesting a facilitating role of ADORA2A in the VEGF pathway, the molecular target of current treatments for diabetic eye diseases. Over the past decade, ADORA2A antagonists have been generated to treat Parkinson’s disease and have shown a good safety profile^12^. All these circumstances collectively indicate that targeting ADORA2A holds significant promise in the treatment of pathological angiogenesis in vision-threatening eye diseases and many other diseases highly dependent on pathological angiogenesis.

## Methods

### Chemicals and reagents

Recombinant hVEGF165 was from R&D Systems (Minneapolis, MN, USA). Collagen type 1 (rat tail) was from BD Biosciences (Erembodegem, Belgium). Adenosine, erytho-9-(2-hydroxy-3-nonyl) adenine (EHNA), cobalt chloride (CoCl_2_), and dimethyl sulfoxide were from Sigma-Aldrich (Bornem, Belgium). Calcein-AM, l-glutamine, and penicillin/streptomycin were from GIBCO (Grand Island, NY), and 4, 6,-diamidino-2-phenylindole (DAPI) was from Invitrogen (Invitrogen, Life Technologies, Ghent, Belgium). The PFKFB3 inhibitor 3-(3-pyridinyl)-1-(4-pyridinyl)-2-propen-1-one (3PO), the glycolysis inhibitor 2-deoxy-d-glucose (2-DG), and the γ-secretase inhibitor *N*-[*N*-(3,5-Difluorophenacetyl)-l-alanyl]-S-phenylglycine t-butyl ester (DAPT) were from Merck Millipore (Overijse, Belgium).

### Mouse generation and breeding

Animals were used according to the National Institutes of Health Guide for the Care and Use of Laboratory Animals and in accordance with the protocol approved by the Institutional Animal Care and Use Committee at the Augusta University. The floxed *Adora2a* (*Adora2a* ^flox/flox^) mice were provided by Dr Joel Linden (La Jolla Institute for Allergy and Immunology, La Jolla, California, USA). Cell-specific inactivation of *Adora2a* in ECs or in macrophages was achieved by cross-breeding *Adora2a* ^flox/flox^ mice with Cdh5-Cre transgenic mice (The Jackson Laboratory, Stk#006137, Bar Harbor, ME) or Lysm-cre transgenic mice (The Jackson Laboratory, Stk#004781), respectively. Global homozygous *Adora2a* (*Adora2a* ^*−/−*^) knockout mice were generated as previously described^54^. All mice were on a C57BL/6J background.

### Mouse model of OIR

The OIR model was described previously^55^. Briefly, seven-day-old (P7) C57BL/6J mouse pups, including both males and females, along with the foster/nursing mothers, were exposed to 70% or 75% O_2_ for 5 days to induce vaso-obliteration. At P12, the mice were returned to room air (RA, 21% O_2_) to induce retinal neovascularization, which was maximal at P17. Age-matched mice kept in RA throughout postnatal development (P0-P17) served as the RA controls. Underdeveloped neonatal mice with weight less than 6 g at P17 were excluded.

### Laser-capture microdissection of retinal vessels

Retinal vessels were microdissected with laser capture in retinal cross sections from *Adora2a* ^flox/flox^Cdh5^cre^ (*Adora2a* ^*VEC-KO*^) and *Adora2a* ^flox/flox^ (*Adora2a* ^*WT*^) mice at postnatal day (P)17, as described previously^2, 56^. In brief, eyes were embedded in OCT and flash frozen immediately following enucleation. Eyes were cyrosectioned under RNase free conditions into 10-μm sections, and collected on RNase-free polyethylene naphthalate glass slides (11505189, Leica). Sections were dehydrated with 70, 90, and 100% ethanol washes and stained with isolectin (1:50 in 1 mM CaCl_2_). Retinal vessels were microdissected with a Leica LMD 6000 system (Leica Microsystems) and collected directly into RNA stabilizing buffer from the RNeasy Micro kit (Qiagen, Chatsworth, CA). RNA was extracted from microdissected tissues using the RNeasy kit as described above (Qiagen), and Real-Time PCR was performed with the generated cDNA.

### Isolation of mouse retinal endothelial cells

Isolation of MAECs was performed according to protocols as described previously with some modifications^57^. Briefly, eyes from one litter (5 to 6 pups) of OIR-*Adora2a* ^*VEC-KO*^ and *Adora2a* ^*WT*^mice at P17 were enucleated and hemisected. The retinas were dissected out and kept in pre-cooling phosphate-buffered saline (PBS) buffer. Retinas (10 to 12 from one litter) were pooled together, rinsed with PBS buffer, quickly minced into small pieces in a 1.5 ml tube using eye scissors, and digested in 8 ml of collagenase type II (Worthington, 2 mg/ml in serum free DMEM, Corning, NY, USA) for 20 min at 37 °C. Following digestion, DMEM with 20% FBS was added and cells were pelleted. The cellular digests then were filtered through 70-μm and 40-μm nylon filters (Corning, NY, USA), centrifuged at 500×*g* for 5 min at 4 °C to pellet cells, and cells were washed with pre-cooling PBS containing 0.5% bovine serum albumin (BSA). The cells were resuspended in 100 μl pre-cooled PBS containing 0.5% BSA and 2 mM EDTA, and incubated with CD31-MicroBeads for 10 min at 4 °C (Miltenyi Biotec Inc). After affinity binding, CD31-positive cells were obtained via magnetic separation using a MACS separator (Miltenyi Biotec). Purified ECs were immediately lysed for Real-Time PCR assay.

### Cell culture and treatments

Human primary retinal microvascular ECs (HRMECs) were obtained from Cell Biologics (Cat. No. H-6065; Chicago, IL, USA) and used between passages 3-8. The type of cells and no pathogen (including mycoplasma) contamination were confirmed by the supplier.

HRMECs were cultured in Vessel Cell Basal Medium (VCBM, ATCC, Manassas, VA, USA) supplemented with Microvascular Endothelial Cell Growth Kit-BBE (ATCC), and 1% penicillin/streptomycin, or in Complete Human Endothelial Cell Medium (Cell Biologics). In some experiments, HRMECs were incubated with 20 ng/ml recombinant hVEGF, 10 μM DAPT, 200 μM CoCl_2_, or 20-100 µM adenosine in the presence of 10 µM EHNA according to the protocol previously described^58, 59^. For the experiments requiring hypoxia, HRMECs were placed in a modular incubator chamber (Thermo Scientific, Waltham, MA) with 0.1–2% O_2_.

### Adenoviral transduction of HRMECs

Ad-A_2A_R, a recombinant adenovirus vector expressing human *ADORA2A*; Ad-mutHIF-1α encoding the mutant human HIF-1α construct containing mutations at P564A and N803A; and Ad-mutHIF-2α containing mutations at P531A and N847A, were generated as previously described^60^. Expressed mutHIF-1α and mutHIF-2α are stable and constitutively active under normoxic conditions^60^. Adenovirus encoding the inert *Escherichia coli LacZ* gene was used as a negative control (Ad-Ctrl). These adenoviruses were expanded in HEK293 cells, and the virus concentration was determined using an Adeno-XTM rapid titer kit (Clontech). Adenoviral infection of HRMECs were carried out at a multiplicity of infection of 10 pfu per cell, as described previously^60^.

### RNA interference

HRMECs were transfected at 60–70% confluence with 30 nM siRNAs targeting human *ADORA2A* (Adenosine A2A-R siRNA (siA_2A_R), Cat. No. sc-39850; Santa Cruz Biotechnology, Dallas, Texas, USA) or with a non-targeting negative control (Control siRNA-A (siCtrl), Cat. No. sc-37007; Santa Cruz Biotechnology) using siRNA transfection reagent (Santa Cruz Biotechnology) or Lipofectamine RNAiMAX Reagent (Invitrogen) per the manufacturer’s protocol. Knockdown of HIF-1α and HIF-2α in HRMECs was carried out by using predesigned SmartPool siRNA purchased from Dharmacon (siHIF-1α, Cat. No. L-004018; siHIF-2α, Cat. No. L-004814; Lafayette, CO). Twenty-four hours after transfection, the medium was changed to fresh complete VCBM, and cells were maintained for an additional 24 h before further experiments.

### Spheroid capillary sprouting assay

HRMECs (750 cells) were incubated overnight in 25% VCBM (25% VCBM complete medium + 75% VCBM basal medium) containing 0.25% (w/v) methylcellulose (Sigma-Aldrich) to form spheroids as described previously^61, 62^. To assess tip cell competition, cells were mixed at a 1:1 ratio. After 24 h, spheroids were harvested and embedded in 0.9 ml collagen solution in pre-warmed 24-well plates, with a final concentration of rat type I collagen (BD Biosciences) at 1.5 mg/ml. The spheroid-containing gels were rapidly transferred into a humidified incubator (37 °C, 5% CO_2_) and allowed to polymerize (20 min) after which 0.1 ml VCBM basal medium was pipetted on top of the gel containing the corresponding cytokines or compounds. After 24 h, cells were fixed with pre-warmed 4% paraformaldehyde (PFA), stained with DRAQ5 (Thermo Scientific) to mark EC nuclei and imaged using a Zeiss LSM 780 Inverted Confocal Microscope. The number of sprouts and cumulative length of sprouts per spheroid were quantified from 10 spheroids for each condition using Image J software.

### Fluorescence immunostaining in whole-mount retinas

OIR**-**mice at P17 were euthanized and perfused successively with PBS and 4% PFA, and the intact retinas were collected. Retinas were blocked and permeabilized in PBS containing 10% goat serum and 1% Triton-X-100 (Sigma-Aldrich) for 30 min. Endogenous Fc receptors and IgG were blocked with rat anti-mouse CD16/CD32 (Mouse BD Block^TM^, 1:50, BD Biosciences, 553142) and the blocking reagent provided in the mouse-on-mouse kit (Vector Laboratories, Cat. No. FMK-2201, Burlingame, CA, USA), respectively. Retinas were then incubated with primary antibodies against mouse Adora2a (1:100, Millipore, 05-717), rabbit IBa1 (1:400, Sakura Finetek, 019-19741, Torrance, CA), and Alexa488- or Alexa-594 labeled *Griffonia simplicifolia* isolectin B4 (1:200, Invitrogen, 121411 and 121413, Carlsbad, CA, USA) overnight at 4 °C, followed by incubation with fluorescence-conjugated cross-adsorbed secondary antibody (1:500, Molecular Probes, Life Technologies, A-21131, Carlsbad, CA,USA) for 1 hour, and then counterstained with DAPI (Invitrogen). Retinas were flat mounted on microscope slides in mounting medium (Vectashield; Vector Laboratories) and examined by confocal microscopy (Zeiss 780; Carl Zeiss, Jena, Germany). Areas of vaso-obliteration and vitreoretinal neovascular tufts were quantified using Adobe Photoshop CS 5 software.

### Immunofluorescence of eye sections

Eyes were fixed in 4% PFA for 2 h at room temperature and equilibrated in 30% sucrose at 4 °C, followed by embedding in OCT. Sections (10-μm thick) were heated at 98 °C for 10 min in citric acid buffer for antigen retrieval, blocked with 10% goat serum for 1 hour, and incubated with mouse HIF-1α (1:100, BD Biosciences, 610958), rabbit Ki-67 (1:200, RM-9106, Thermo Scientific), rabbit PFKFB3 (1:100, Proteintech, 13763-1-AP), rat CD31 (1:25, Invitrogen, DIA-310) and/ or Alexa-594 labeled *Griffonia simplicifolia* isolectin B4 (1:100, Invitrogen, Cat. No. 121413) overnight at 4 °C, followed by incubation with fluorescence-conjugated secondary antibody (1:250, Molecular Probes, Life Technologies, Carlsbad, CA,USA) for 1 hour. For Ki-67/ ERG double immunofluorescent staining, sections were then stained with Anti-ERG antibody (Alexa Fluor® 594) (1:200, Abcam, Clone number: EPR3864) overnight at 4 °C. Sections were washed with PBS, immersed in ProLong Gold mounting medium with DAPI (Invitrogen) to visualize the nuclei, and examined using confocal microscopy. For all immunofluorescence experiments, parallel groups of sections were stained with only primary or secondary antibody as negative controls.

### Neovascular nuclei quantification

To quantify neovascular nuclei, retina sections of OIR mice were stained with hematoxylin-eosin (H&E). The extent of neovascularization was evaluated by counting the number of neovascular nuclei, which were defined as the nuclei of cells that extended beyond the inner limiting membrane of the retina into the vitreous. In this study, eyes of 6–8 mice from each group were examined and analyzed. Neovascular nuclei were counted in cross-sections with light microscopy under 40× magnification by an investigator who was blinded to the specific group assignment.

### Real-Time PCR analysis

Total RNA of HRMECs and retinas were extracted using Trizol Reagent (Invitrogen, Grand Island, NY). In all, 0.1–1.0 μg sample of total RNA was utilized as a template for reverse transcription using the QuantiTect Reverse Transcription Kit (QIAGEN) for HRMECs or iScriptTM cDNA synthesis kit (Bio-Rad) for retinas. Real-time PCR was performed on a StepOne Plus System (Applied Biosystems, Grand Island, NY) using Power SYBR GreenMaster Mix (Applied Biosystems) with the respective gene-specific primers listed in Supplementary Table 1. Quantification of relative gene expression was calculated with the efficiency-corrected 2^−△△CT^ method using GUBS and 18S rRNA (for human RNA), or HMBS and HRPT (for mouse RNA) as the internal control, and data were presented as fold change relative to control groups.

### Protein extraction and western blot

HRMECs were lysed with RIPA buffer (Fisher) supplemented with 1% proteinase inhibitor cocktail (Pierce, Rockford, IL) and 1% phosphatase inhibitors (Pierce). After sonication and centrifugation of cell lysates, protein was quantified with the BCA assay and then loaded in the 8% sodium dodecyl sulfate polyacrylamide gel electrophoresis (SDS-PAGE) gel at 10–20 μg per lane. Primary antibodies used in this study were as follows: ADORA2A (Sigma, A-269; rabbit, 1:500), ADORA2A (Millipore, 05-717; Mouse, 1:1000), HIF-1α (BD Biosciences, 610958; mouse, 1:500), HIF-1α (R&D Systems, AF1935; goat, 1:1000), HIF-2α (Novus Biologicals, NB100-122; Littleton, CO, USA; rabbit, 1:1000), PFKFB3 (Proteintech, 13763-1-AP; rabbit, 1:2000), p-AKT (Cell Signaling Technology, 4060; rabbit, 1:2000), AKT (Cell Signaling Technology, 4691; rabbit, 1:2000), p-ERK1/2 (Cell Signaling Technology, 4370; rabbit, 1:2000), ERK1/2 (Cell Signaling Technology, 4695; rabbit, 1:2000), p-p38 (Cell Signaling Technology, 9215; rabbit, 1:1000), p38 (Cell Signaling Technology, 8690; rabbit, 1:1000), p-JNK1/2 (Cell Signaling Technology, 9251; rabbit, 1:1000), JNK1/2 (Cell Signaling Technology, 9252; rabbit, 1:1000), p-p70^S6K^ (Cell Signaling Technology, 9234; rabbit, 1:1000), p70^S6K^ (Cell Signaling Technology, 2708; rabbit, 1:1000), p-eIF-4E (Cell Signaling Technology, 9741; rabbit, 1:1000), eIF-4E (Cell Signaling Technology, 2067; rabbit, 1:1000), and β-actin (Cell Signaling Technology, 3700; mouse, 1:5000). Images were taken with the ChemiDoc MP system (Bio-Rad), and band densities were quantified using Image Lab software (Bio-Rad). Uncropped scans for western blots are provided in Supplementary Figs. 16–18.

### Capillary tube network formation

HRMECs were seeded on growth factor-reduced Matrigel (BD Bioscience)-coated 96-well plates (1 × 10^4^ cells per well) in 0.1 ml VCBM for 4 h. The endothelial tubule formation was observed and photographed using an inverted confocal microscope after staining with Calcein AM. Cumulative tube length was quantified using the Image J software. Branch points were manually counted.

### Metabolic measurements

HRMECs were seeded on Seahorse XF96 polystyrene tissue culture plates (Seahorse Bioscience, North Billerica, MA), and incubated at 37 °C overnight in 25% VCBM. To avoid differences due to unequal cell numbers and growth rates, all measurements were made starting with confluent cells by seeding 1.5 × 10^4^ per well. The next day, the medium was changed to XF base Medium (Seahorse Bioscience) supplemented with 2 mM glutamine (for ECAR), or supplemented with 25 mM glucose, 1 mM pyruvate, and 2 mM glutamine (for oxygen consumption rate, OCR), and then the plate was incubated for 1 h in a non-CO_2_ incubator at 37 °C. ECAR and OCR were measured with an XF^e^96 extracellular flux analyzer (Seahorse Bioscience). Inhibitors and activators were used in these tests at the following concentrations: glucose (10 mM), oligomycin (2 µM), 2-DG (50 mM), FCCP (1 µM), antimycin A (0.5µM), and rotenone (0.5 µM).

### Lactate measurements

The levels of secreted lactate in cell medium of HRMECs were determined using the Lactate Assay Kit (Sigma-Aldrich, Cat. No. MAK064).

### WST-1 proliferation assay

HRMECs were seeded at 4 × 10^3^ cells per well in 96-well plates. The cells were incubated in 25% VCBM for 72 h under normoxia (21% O_2_) or hypoxia (0.5% O_2_) and proliferation was assessed by WST-1 assay (Sigma-Aldrich, Cat. No. 5015944001).

### Ki-67 staining and BrdU incorporation analysis

HRMECs were treated with BrdU labeling reagent (Invitrogen) for 16 h. Following BrdU treatment, cells were fixed with 4% PFA for 10 min, permeabilized in PBS containing 0.5% Triton-X-100 for 15 min, treated with 2N HCl for 30 min, blocked with 10% goat serum for 1 h, and then incubated with a mouse monoclonal anti-BrdU antibody (1:200, Invitrogen, Cat. No. 03-3900) and rabbit anti-Ki-67 antibody (1:200, Thermo Scientific, Cat. No. RM-9106) overnight at 4 °C, followed by incubation with fluorescence-conjugated secondary antibody (1:250, Molecular Probes, Life Technologies, Carlsbad, CA, USA) for 1 h. The cells were then immersed in ProLong Gold mounting medium with DAPI (Invitrogen) to visualize the nuclei. Images were obtained using an inverted fluorescence microscope (Zeiss Axio Observer Z1) or upright confocal microscope (Zeiss 780; Carl Zeiss). The number of Ki-67 or BrdU-positive cells was counted in six non-overlapping and randomly selected microscopic fields per slide.

### Statistical analysis

The optimal animal numbers and sample sizes were estimated based on power analysis, prior experience, and our preliminary data. Grouping was performed in a randomized manner when using C57BL/6J wild-type mice. No randomization was used when using other mice, since all these mice were genetically defined, inbred mice. Data analysis for in vivo angiogenic phenotype was performed in a blinded fashion. Data are presented as means ± s.e.m. Statistical analysis was performed using GraphPad Prism Software (La Jolla, CA). After the normal distribution was confirmed with the Kolmogorov–Smirnov test, statistical comparisons were done using two-tailed unpaired Student’s *t*-test or one- or two-way analysis of variance (ANOVA) followed by Bonferroni’s post hoc tests when appropriate. Two-sided *P*-values were calculated and *P* < 0.05 denoted significance. Statistical significance was defined as follows: *P < 0.05, **P < 0.01, ***P < 0.001.

### Data availability

The authors state that all relevant data are available within the article and its Supplementary Information files or are available from the corresponding authors upon reasonable request.

## Electronic supplementary material

Supplementary Information
