# Supplementary material for: Endothelial adenosine A2a receptor-mediated glycolysis is essential for pathological retinal angiogenesis
Source: Nat Commun. 2017 Sep 19;8:584. doi: 10.1038/s41467-017-00551-2 (PMC5605640; doi:10.1038/s41467-017-00551-2)
Supplement: Supplementary file 1 — Supplementary Information [file 41467_2017_551_MOESM1_ESM.pdf]

File Name: Supplementary Information
Description: Supplementary Figures and Supplementary Table.

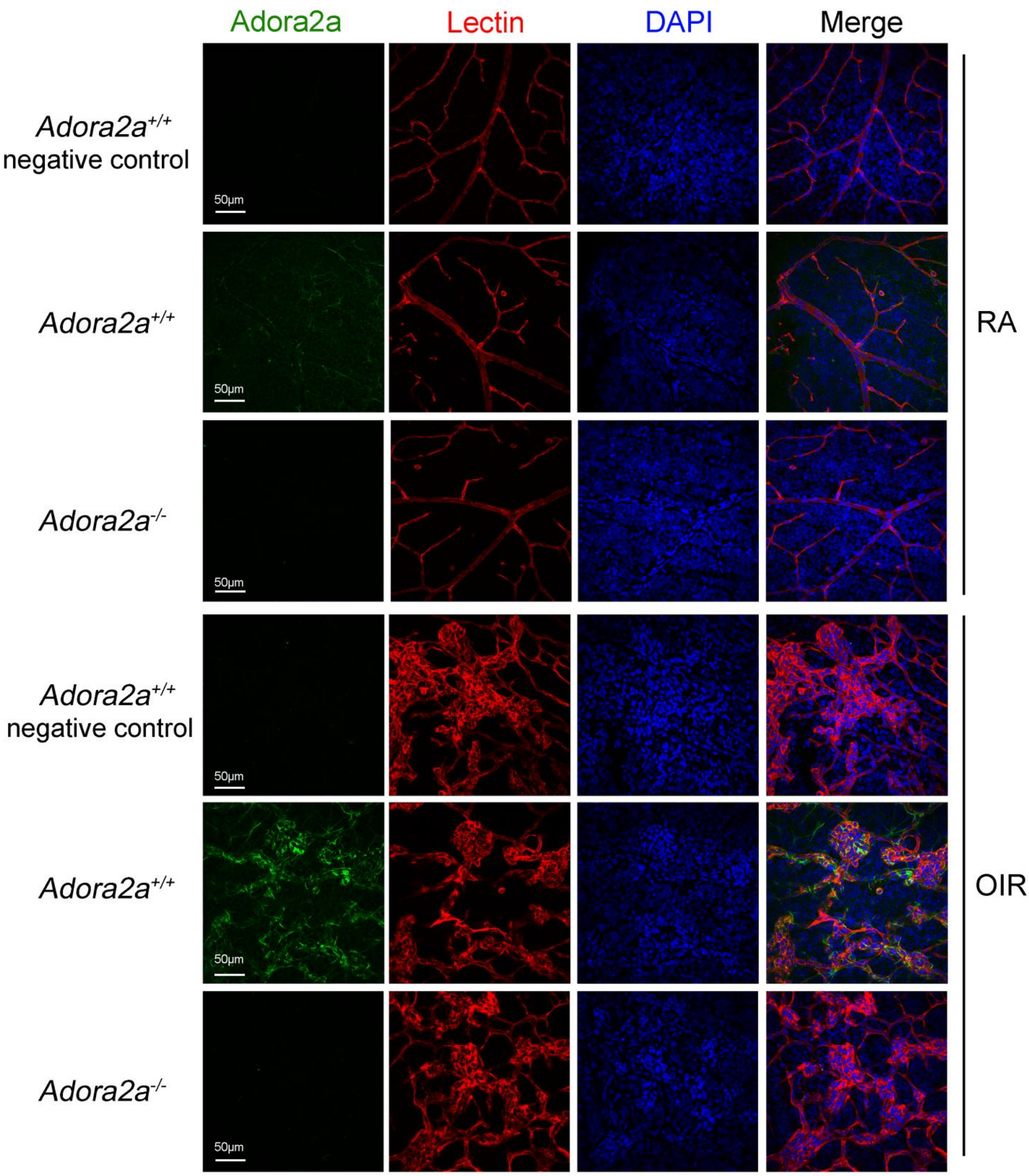

Supplementary Figure 1. Ablation of adenosine A2a receptor expression in global homozygous *Adora2a* knockout (*Adora2a* $^{-/-}$) mice, complemented to Fig 1e. P17 RA and OIR retinas from wild-type (*Adora2a* $^{+/+}$) and *Adora2a* $^{-/-}$ mice were stained with anti-Adora2a (green), isolectin B4 (Lectin, red, vessel), and DAPI (blue, nuclei). Negative control groups were processed the same way, except for omission of the primary antibody. Scale bars: 50 μm. n = 3 mice per group.

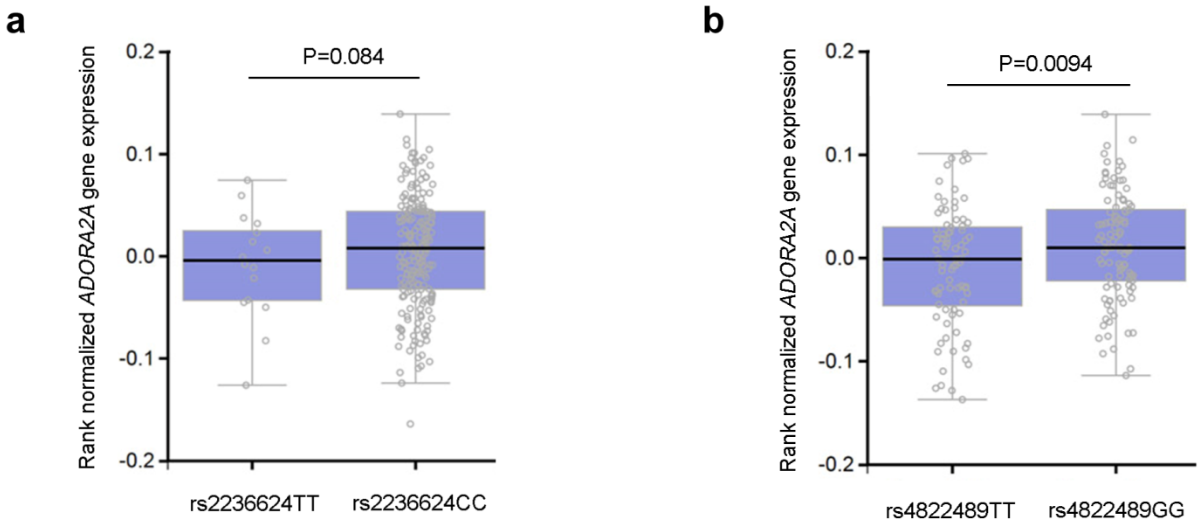

Supplementary Figure 2. ***ADORA2A*** **mRNA levels in whole blood cells in selected human individuals.** (**a**) Levels of blood cell *ADORA2A* mRNA in human individuals homozygous for the T or C alleles of SNP rs2236624. n = 16 for T and 204 for C allele of SNP rs2236624, *P* = 0.084. (**b**) Levels of blood cell *ADORA2A* mRNA in human individuals homozygous for the T or G allele of SNP rs4822489. n = 80 for T and 104 for G allele of SNP rs4822498, *P* = 0.0094. Data are represented as means ± s.d. Statistical significance was determined by unpaired Student's *t*-test.

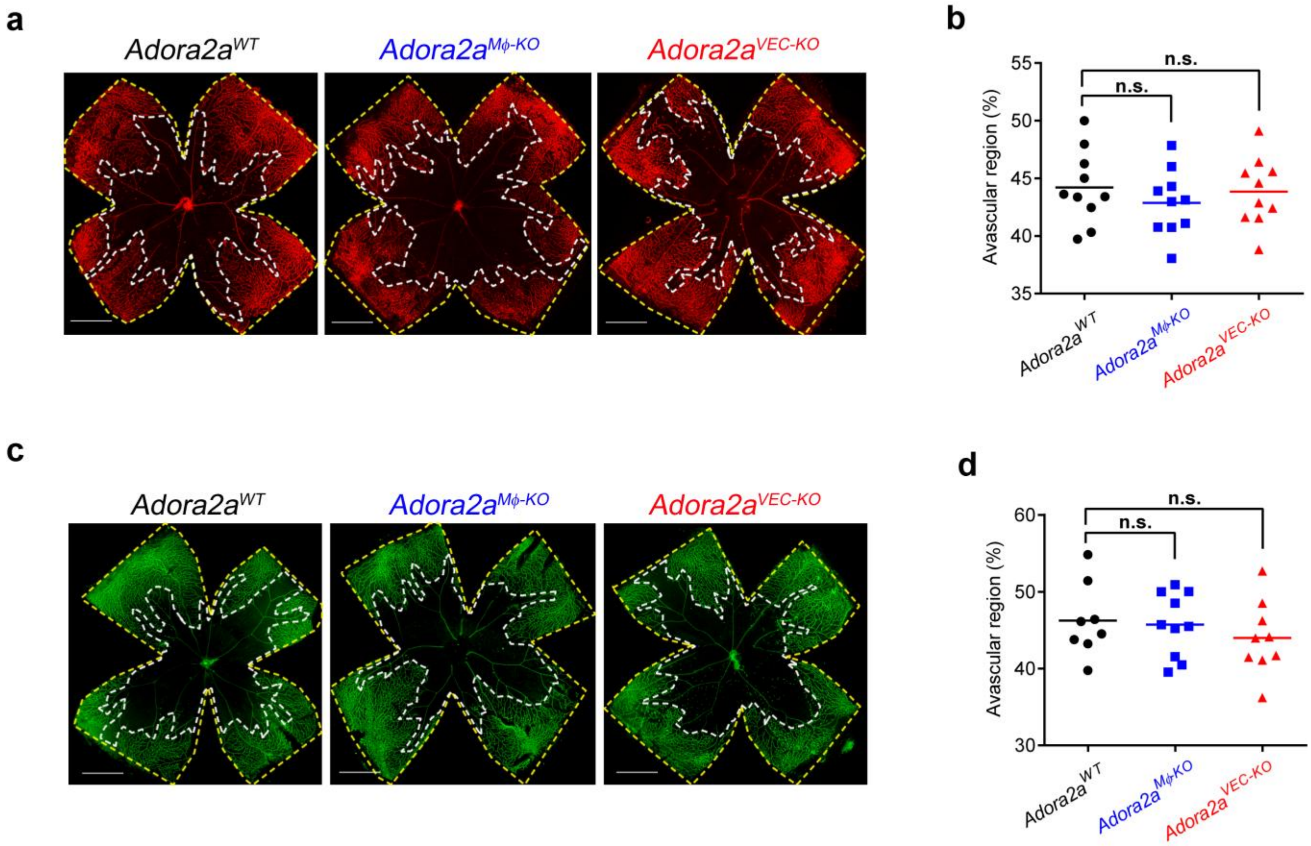

Supplementary Figure 3. Effect of endothelial and macrophage *Adora2a* deficiency on retinal vaso-obliteration at postnatal day (P) 12 in OIR. (**a, c**) Retinal blood vessels of $Adora2a^{WT}$, $Adora2a^{M\varphi\text{-}KO}$ and $Adora2a^{VEC\text{-}KO}$ mice visualized by isolectin B4 staining of OIR retinas at P12 (**a**: 70 % O_2; **c**: 75 % O_2). The entire retinal surface is shown by the yellow dotted line. The vascular area is indicated by the white dotted line. Scale bar: 1000 µm. (**b, d**) Avascular area (%) was quantified as a percentage of the whole retinal surface (n = 8-10 for each group). *P* = NS. Data are represented as means ± s.e.m. Statistical significance was determined by one-way ANOVA followed by Bonferroni test.

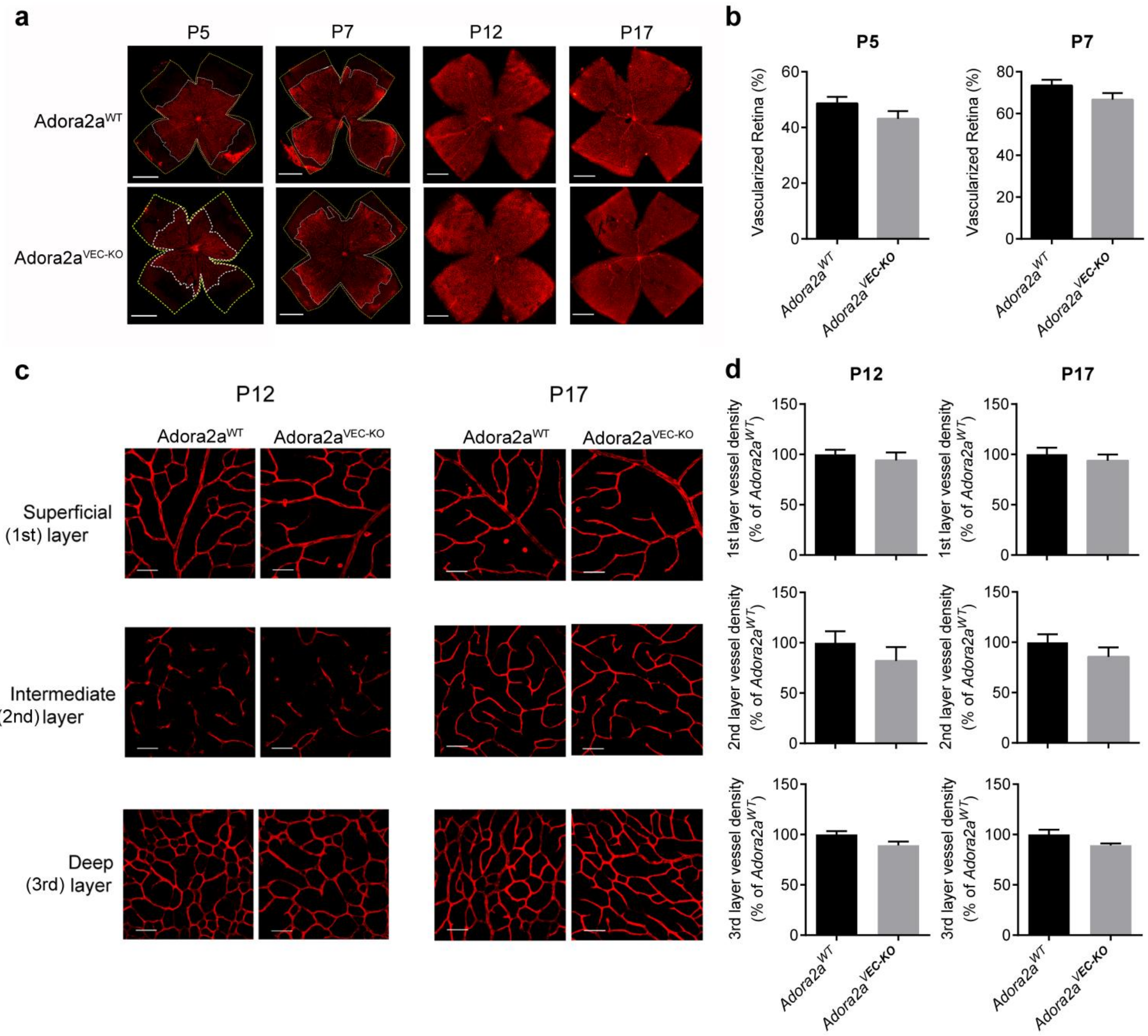

Supplementary Figure 4. Effect of endothelial cell-specific *Adora2a* deficiency on the development of retinal vessels in mice. (**a-b**) The development of retina vasculature of WT and endothelial cell-specific *Adora2a* KO mice at postnatal day (P) 5, P7, P12, and P17 in room air was visualized by isolectin B4 (IB4) staining in whole-mount retinas. Vascularized areas and whole retinal surface are shown by white dotted line and yellow dotted line, respectively. (**b**) The retinal vasculatures of the superficial, intermediate and deep vascular layers were examined at P12 and P17 by IB4 staining of whole-mount retinas. The distributions of three retinal vascular layers were displayed in distinct confocal planes. Scale bar: 50µm. (**c**) Quantification of the superficial vascularized areas of retinas at P5 (n = 6, 8) and P7 (n = 8, 6) as a percentage of the whole retinal area. (**d**) Quantification of the vascular density (vessel coverage area per field) in the superficial layer, intermediate layer, and deep layer at P12 (n = 8, 6) and P17 (n = 6). Scale bar: 50 µm. *P* = NS. Data are represented as means ± s.e.m. Statistical significance was determined by unpaired Student's *t*-test.

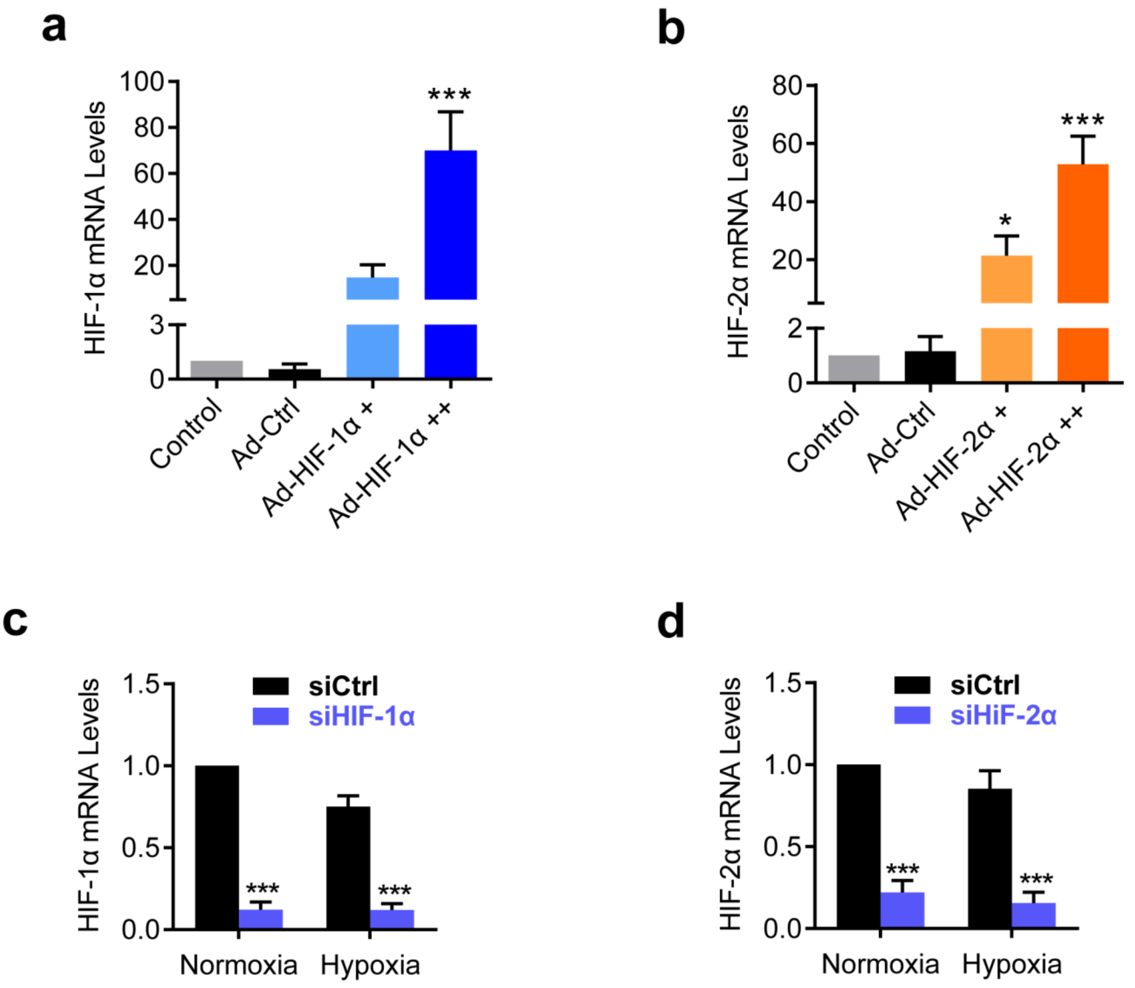

Supplementary Figure 5. Expression of HIF-1α and HIF-2α in HRMECs infected with Ad-mutHIFs or transfected with siHIFs. (**a, b**) Real-Time PCR analysis of mRNA expression of HIF-1α and HIF-2α in HRMECs. Cells were infected with 10 (+) or 30 (++) pfu per cell of either Ad-mutHIF-1α (**a**), Ad-mutHIF-2α (**b**), or Ad-Ctrl. n = 3. *$P < 0.05$, ***$P < 0.001$ vs control. (**c, d**) Real-Time PCR analysis of mRNA levels of HIF-1α and HIF-2α in HRMECs. Cells were transiently transfected with siHIF-1α (**c**), siHIF-2α (**d**), or siCtrl. Forty-eight hours after transfection, cells were exposed to hypoxia (0.5 % O_2) or air (21 % O_2) for an additional 12 h. n = 4. ***$P < 0.001$ vs siCtrl. Data are represented as means ± s.e.m. Statistical significance was determined by unpaired Student's *t*-test.

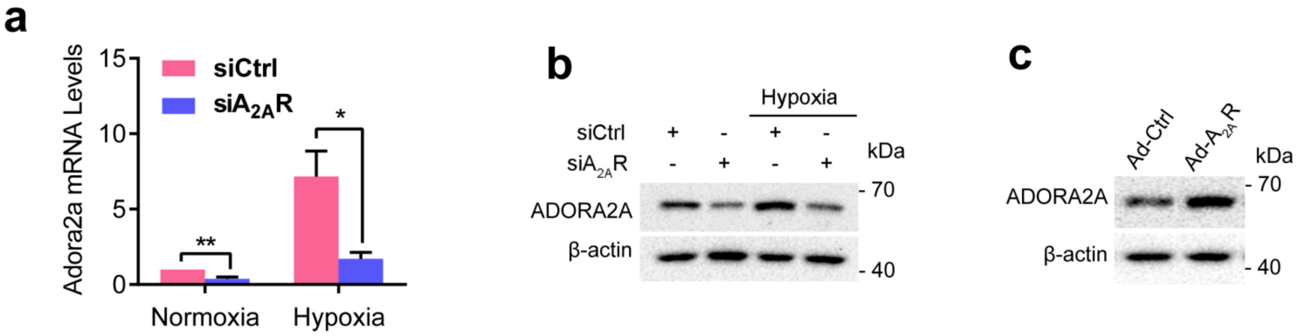

Supplementary Figure 6. Expression of ADORA2A in HRMECs transfected with siA$_{2A}$R or infected with Ad-A$_{2A}$R. (**a, b**) Real-Time PCR (**a**) and Western blot (**b**) analysis of *ADORA2A* mRNA and protein expression in HRMECs transfected with siA$_{2A}$R or siCtrl under hypoxia (0.5 % O_2) or normoxia (21 % O_2). n = 3. *$P < 0.05$, **$P < 0.01$. Data are represented as means ± s.e.m. Statistical significance was determined by unpaired Student's *t*-test. (**c**) Western blot analysis of ADORA2A protein expression in HRMECs infected with Ad-Ctrl or Ad-A$_{2A}$R (10 pfu per cell). n = 3.

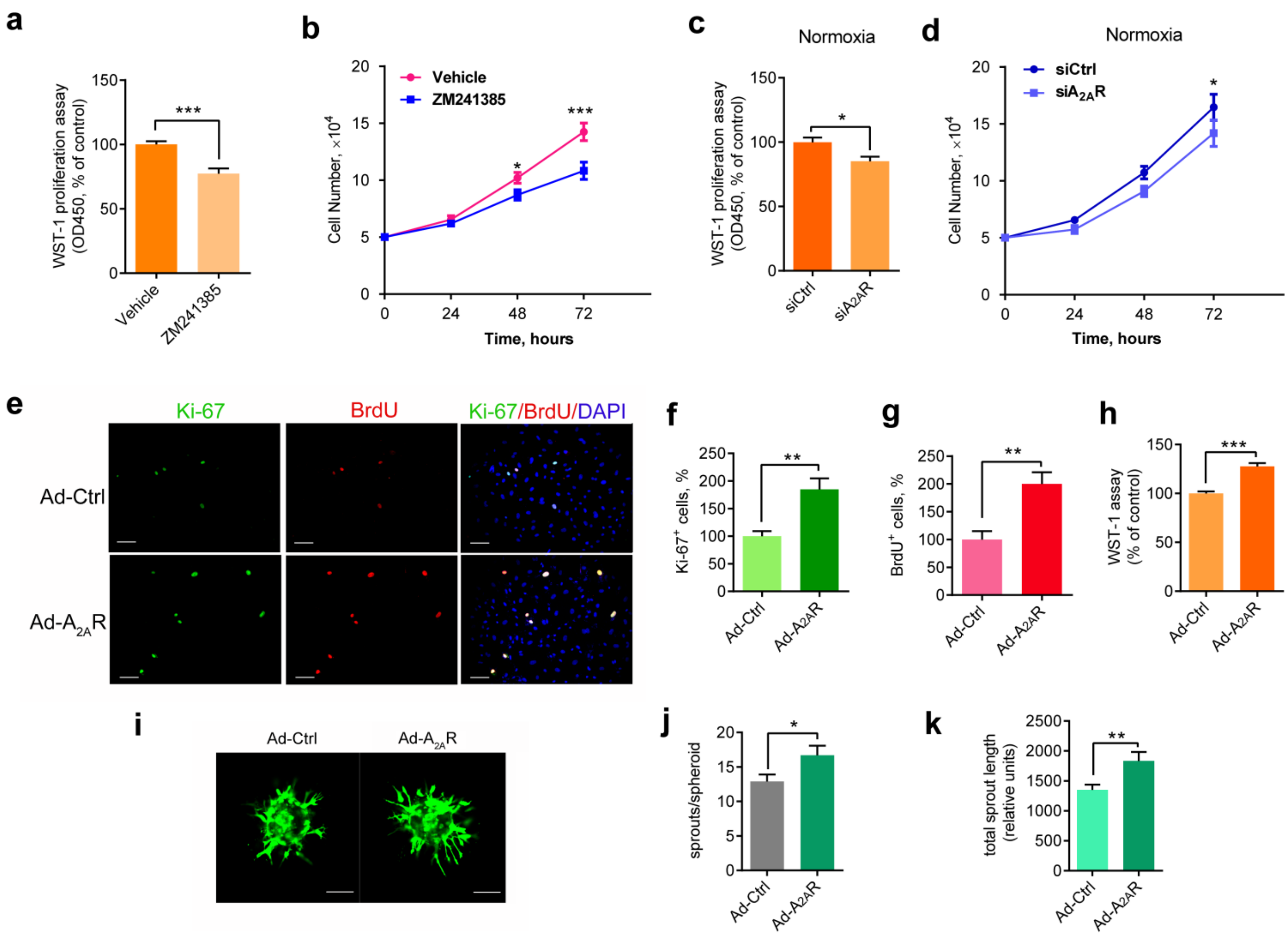

Supplementary Figure 7. Effect of ADORA2A on HRMEC proliferation and sprouting. (**a**) Cell proliferation measured by WST-1 cell proliferation assay. HRMECs were transfected with siA$_{2A}$R or siCtrl under normoxia conditions. n = 6. **P* < 0.05. (**b**) Growth curves of HRMECs over 72 hours. Cells were treated as described in (**a**). n = 6. **P* < 0.05. (**c**) Cell proliferation measured by WST-1 cell proliferation assay. HRMECs were treated with vehicle (DMSO) or ZM241385 (5 µM) for 72 hours under hypoxia conditions (0.5 % O_2). n = 6. ****P* < 0.001. (**d**) Growth curves of HRMECs over 72 hours. Cells were treated as described in (**c**). n = 6. **P* < 0.05, ****P* < 0.001 vs vehicle. (**e-g**) Ki-67, BrdU (bromodeoxyuridine), and DAPI staining of proliferating HRMECs infected with Ad-Ctrl or Ad-A$_{2A}$R in the absence of adenosine. Scale bar: 50µm. n = 6. ***P* < 0.01. (**h**) Cell proliferation measured by WST-1 cell proliferation assay. Cells were infected with Ad-Ctrl or Ad-A$_{2A}$R in the absence of adenosine 72 hours. n = 6. ****P* < 0.001. (**i-k**) Representative images of spheroidal sprouting (**i**) and morphometric quantification of spheroid sprouting by calculating the number of sprouts per spheroid (**j**), as well as total sprout length (**k**). HRMECs were infected with Ad-Ctrl or Ad-A$_{2A}$R, and then were cultured in collagen gel to grow into 3D multicellular spheroids for 24 hours in the absence of adenosine. Scale bar: 100µm. n = 10 per group. **P* < 0.05; ***P* < 0.01. Data are represented as means ± s.e.m. Statistical significance was determined by unpaired Student's *t*-test (for **a,c,f,g,h,j,k**) and two-way ANOVA followed by Bonferroni test (for **b,d**).

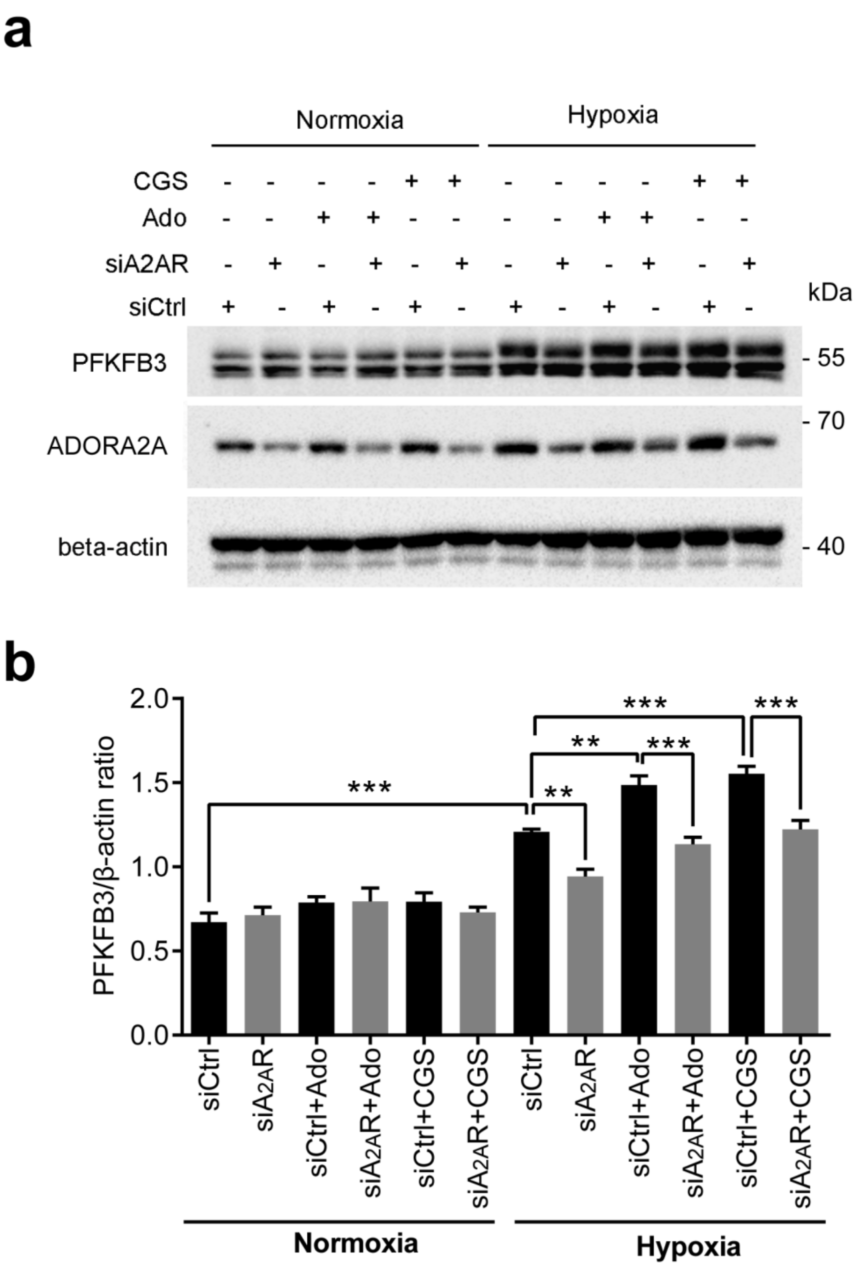

Supplementary Figure 8. Effect of *ADORA2A* knockdown on PFKFB3 protein expression in HRMECs under hypoxia. (**a, b**) Western blot analysis and quantification of PFKFB3 protein expression in HRMECs transfected with siA_{2A}R or siCtrl under hypoxia (0.5 % O_2) or normoxia (21 % O_2), with or without adenosine/ CGS21680 treatment. n = 3. $^{**}P < 0.01$; $^{***}P < 0.001$. Data are represented as means ± s.e.m. Statistical significance was determined by one-way ANOVA followed by Bonferroni test.

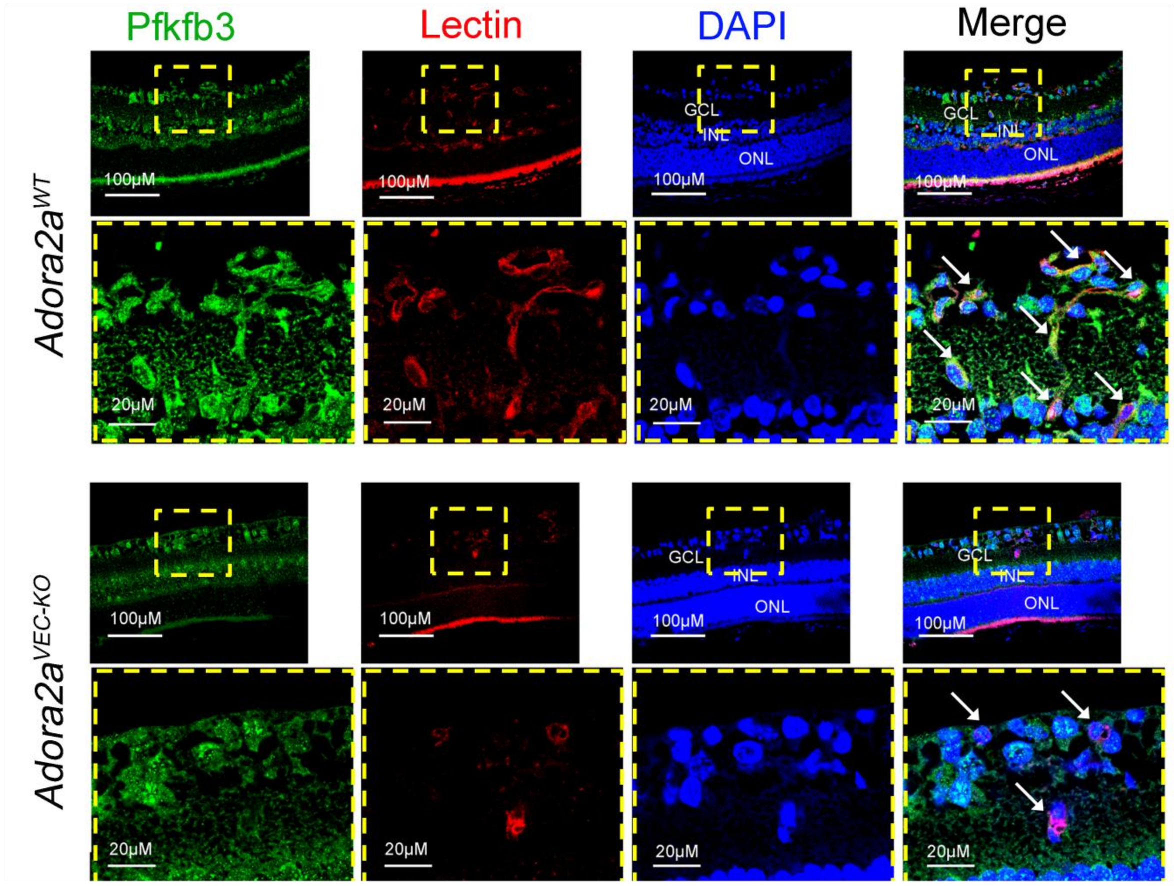

Supplementary Figure 9. Effect of endothelial *Adora2a* deficiency on Pfkfb3 expression in OIR retinas. Pfkfb3 immunofluorescent staining of OIR retinas of *Adora2a*^{WT} and *Adora2a*$^{VEC\text{-}KO}$ mice at postnatal day 17. Representative green (Pfkfb3), red (vessel, Lectin), blue (nuclei, DAPI), and merged images captured with confocal fluorescent microscopy. GCL, ganglion cell layer; INL, inner nuclear layer; ONL, outer nuclear layer.

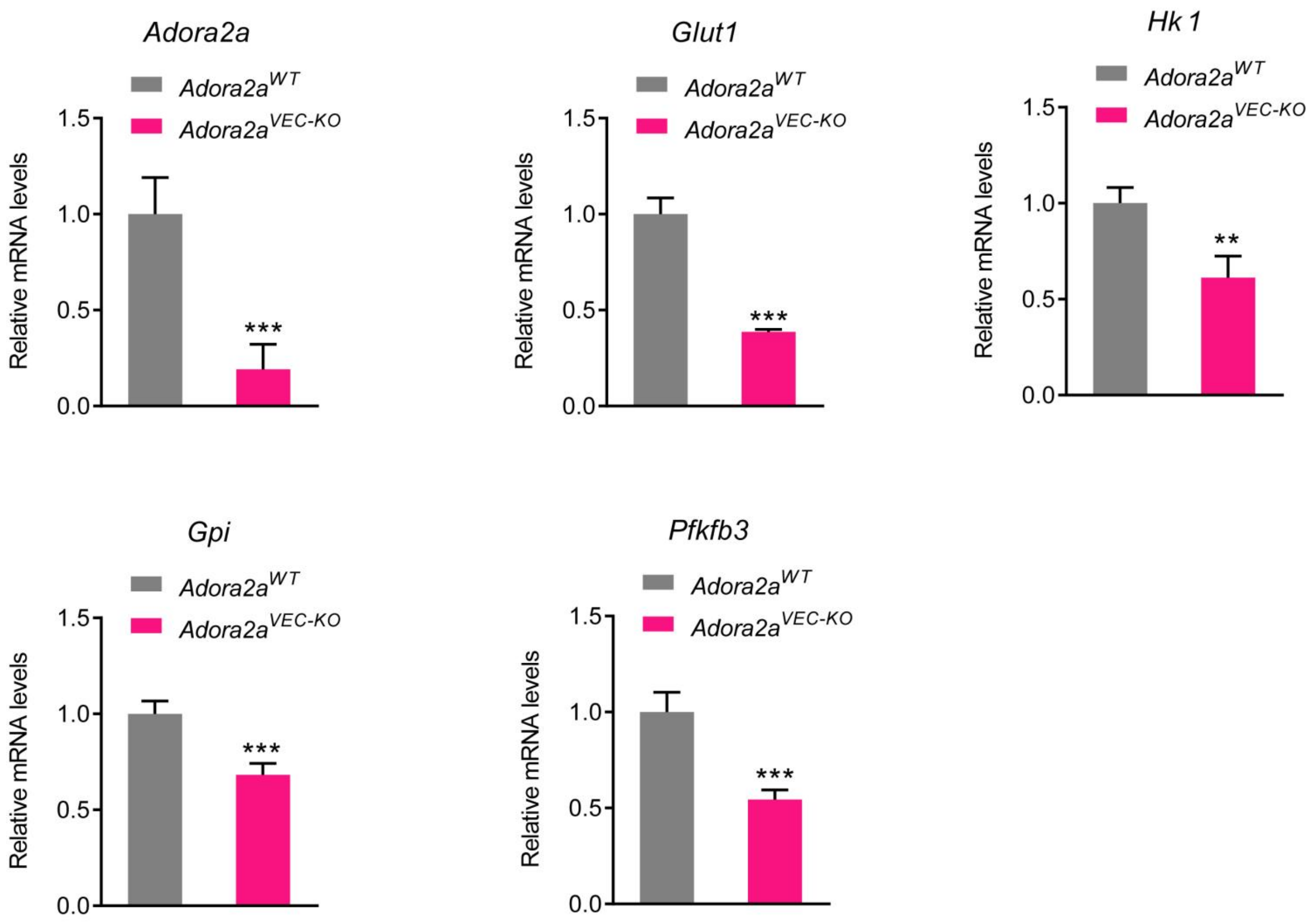

Supplementary Figure 10. Effect of endothelial-specific *Adora2a* deletion on the mRNA expression of the key glycolytic enzymes in mouse retinal ECs from OIR mice. Real-Time PCR analysis of the mRNA levels of *Adora2a*, *Glut1*, *Hk1*, *Gpi*, and *Pfkfb3* in mouse retinal ECs isolated from OIR-*Adora2a*^{WT} and OIR-*Adora2a*$^{VEC\text{-}KO}$ mice at postnatal day 17. n = 4. ***P* < 0.01; ****P* < 0.001 vs *Adora2a*^{WT} group. Data are represented as means ± s.e.m. Statistical significance was determined by unpaired Student's *t*-test.

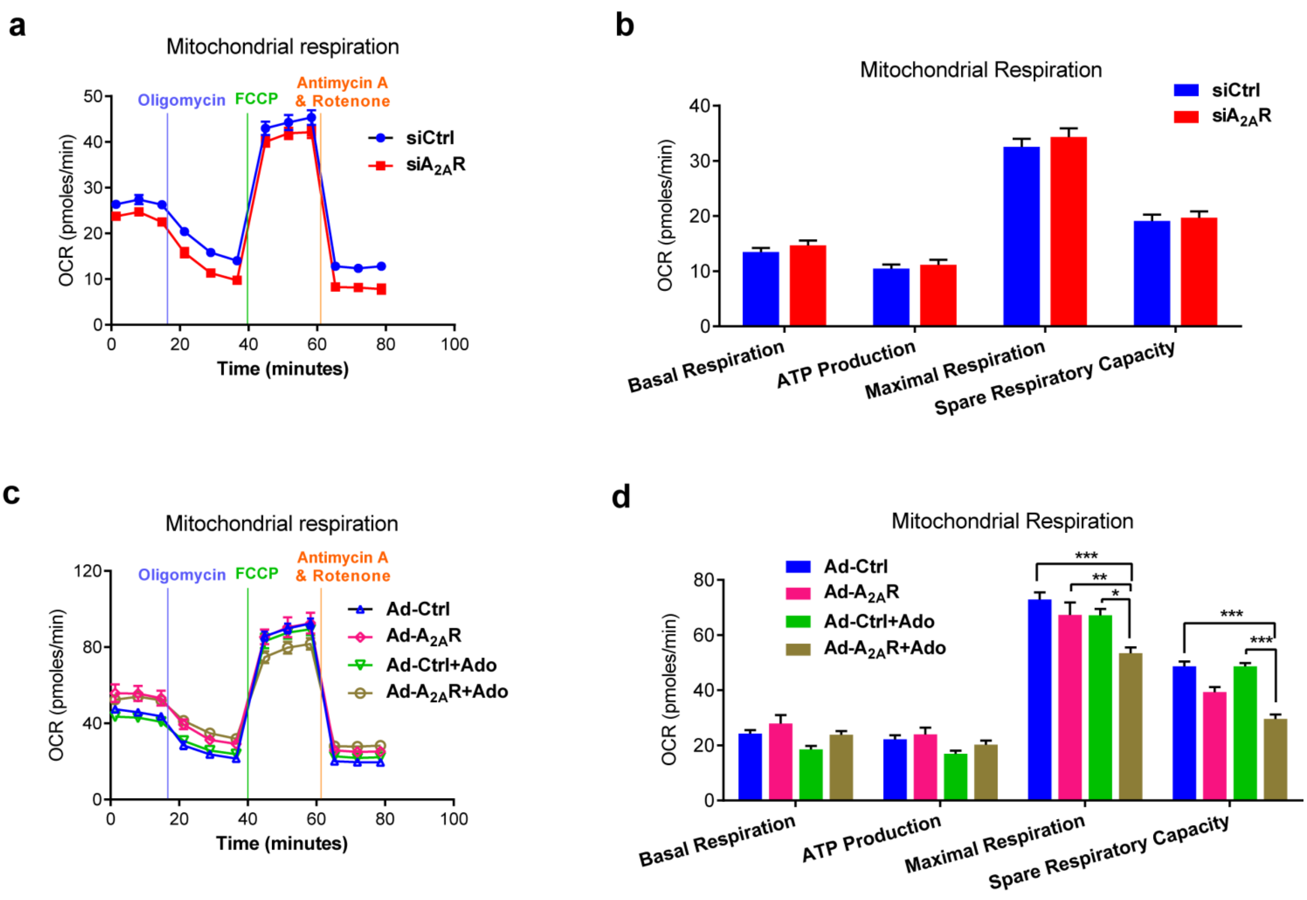

Supplementary Figure 11. Effect of *ADORA2A* knockdown or overexpression on cellular oxygen consumption. OCR profile of HRMECs transfected with siA$_{2A}$R or siCtrl (**a**), or infected with Ad-A$_{2A}$R or Ad- Ctrl in the presence or absence of adenosine (**c**). Vertical lines indicate the time of addition of oligomycin (2 µM), carbonyl cyanide 4-trifluoromethoxy-phenylhy-drazone (FCCP; 1 µM), antimycin A (0.5 µM), and rotenone (0.5 µM). (**b**) and (**d**), Quantification of mitochondrial respiration function parameters of (**a**) and (**c**). n = 8. *P < 0.05, **P < 0.01, ***P < 0.001. Data are represented as means ± s.e.m. Statistical significance was determined by unpaired Student's t-test (for **b**) and one-way ANOVA followed by Bonferroni test (for **d**).

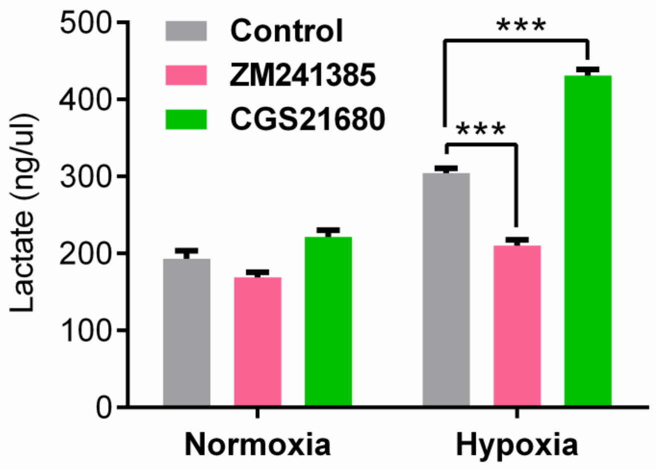

Supplementary Figure 12. Effect of ADORA2A compounds on glycolysis of HRMECs. Levels of secreted lactate of HRMECs treated with ADORA2A antagonist ZM241385 or ADORA2A agonist CGS12680 under normoxia or hypoxia for 24 hours. n = 3. ****P* < 0.001. Data are represented as means ± s.e.m. Statistical significance was determined by one-way ANOVA followed by Bonferroni test.

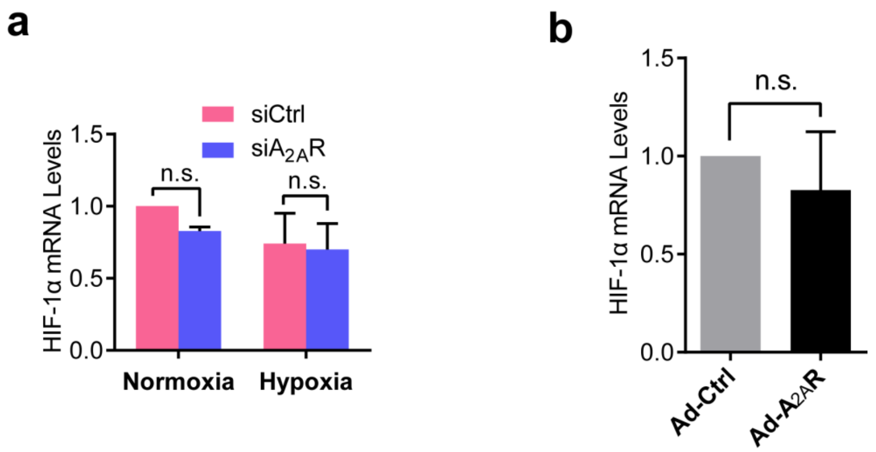

Supplementary Figure 13. (**a-b**) Real-Time PCR analysis of HIF-1α mRNA expression in HRMECs transfected with $siA_{2A}R$ or siCtrl under hypoxia (0.5 % O_2) or normoxia (21 % O_2) (**a**) or infected with Ad-Ctrl or Ad-$A_{2A}R$ under normoxia (**b**). n = 3 for (**a**) and n = 5 for (**b**). Data are represented as means ± s.e.m. Statistical significance was determined by unpaired Student's *t*-test.

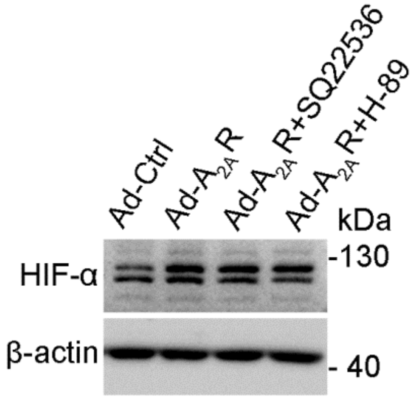

Supplementary Figure 14. Effect of SQ22536 and H-89 treatment on ADORA2A-induced HIF-1α expression in HRMECs. Western blot analysis of HIF-1α expression in HRMECs infected with Ad-A_{2A}R or Ad-Ctrl, in the presence or absence of SQ22536 or H-89. n = 3. Representative images of three independent sets of experiments are shown.

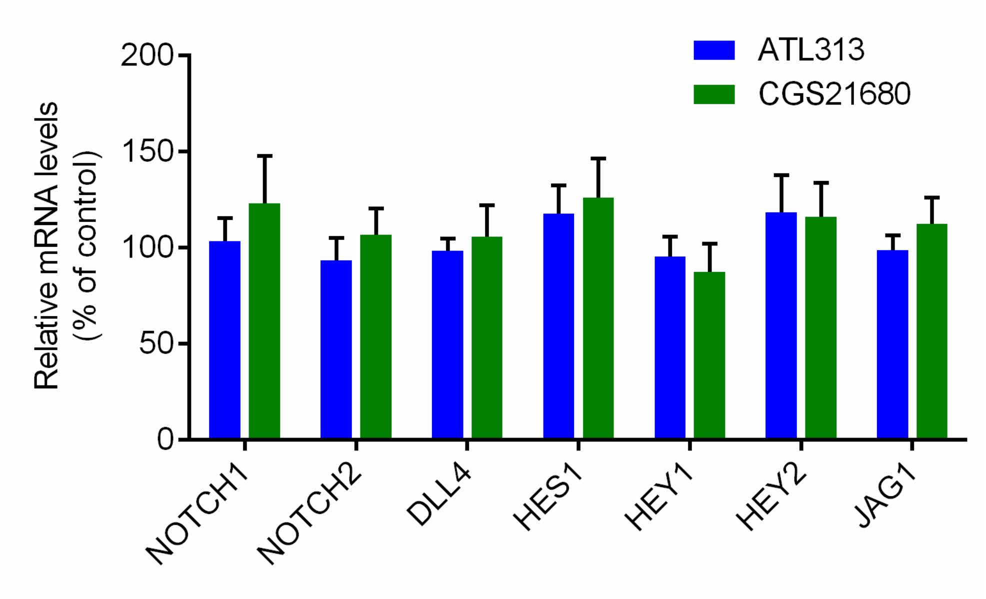

Supplementary Figure 15. Effect of ADORA2A activation on Notch signaling after short-term stimulation by ADORA2A antagonists. Real-Time PCR analysis of the mRNA levels of the notch signaling-related genes, including *NOTCH1*, *NOTCH2*, *DLL4*, *HES1*, *HEY1*, *HEY2*, and *JAG1* in HRMECs treated with ADORA2A agonist ATL313 (100 nM) or CGS12680 (5 μM) for 6 hours. n = 3; *P* = NS. Data are represented as means ± s.e.m. Statistical significance was determined by unpaired Student's *t*-test.

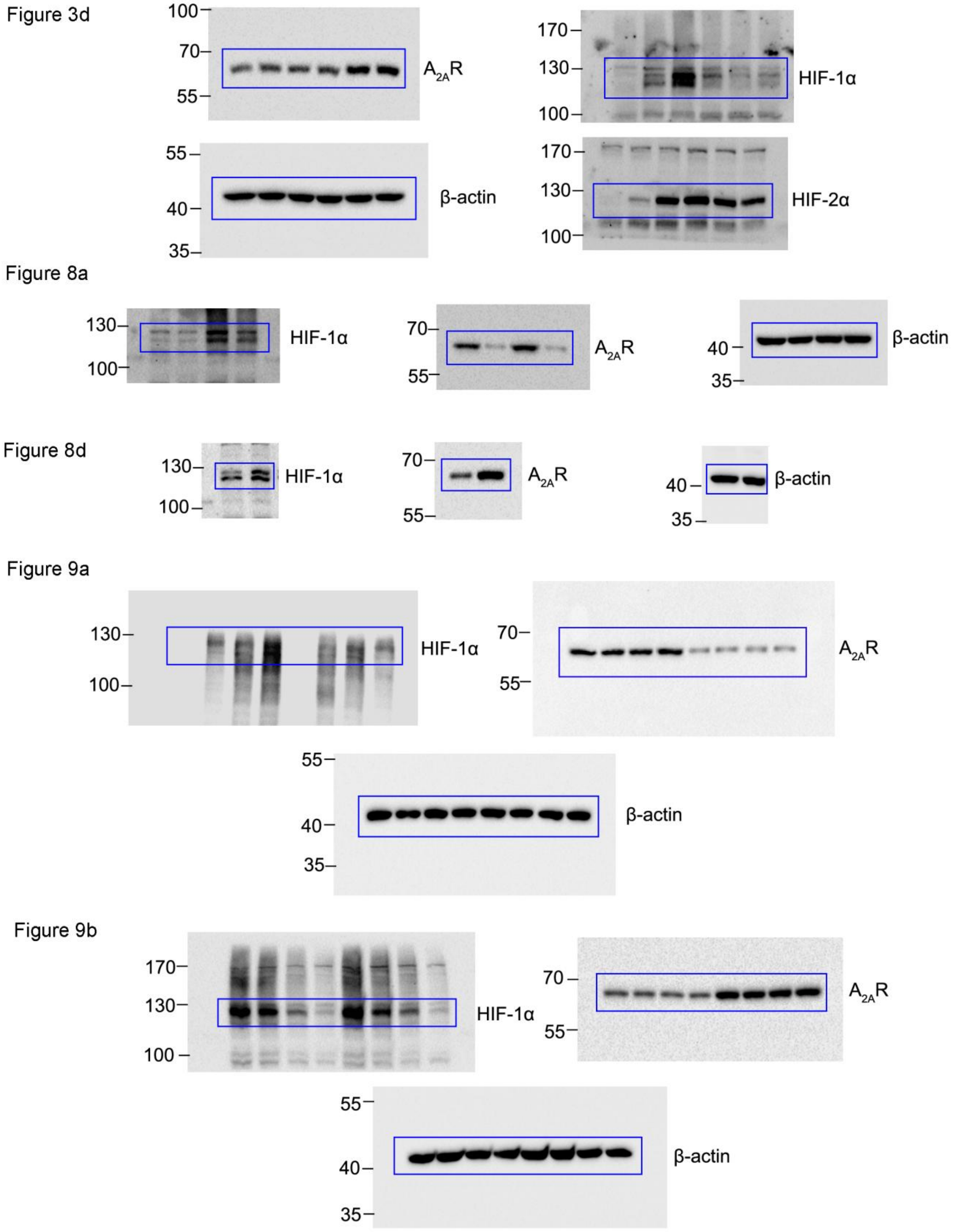

Supplementary Figure 16. Uncropped scans of the Western blots presented in Fig. 3d, 8a, 8d, 9a and 9b.

Figure 9e

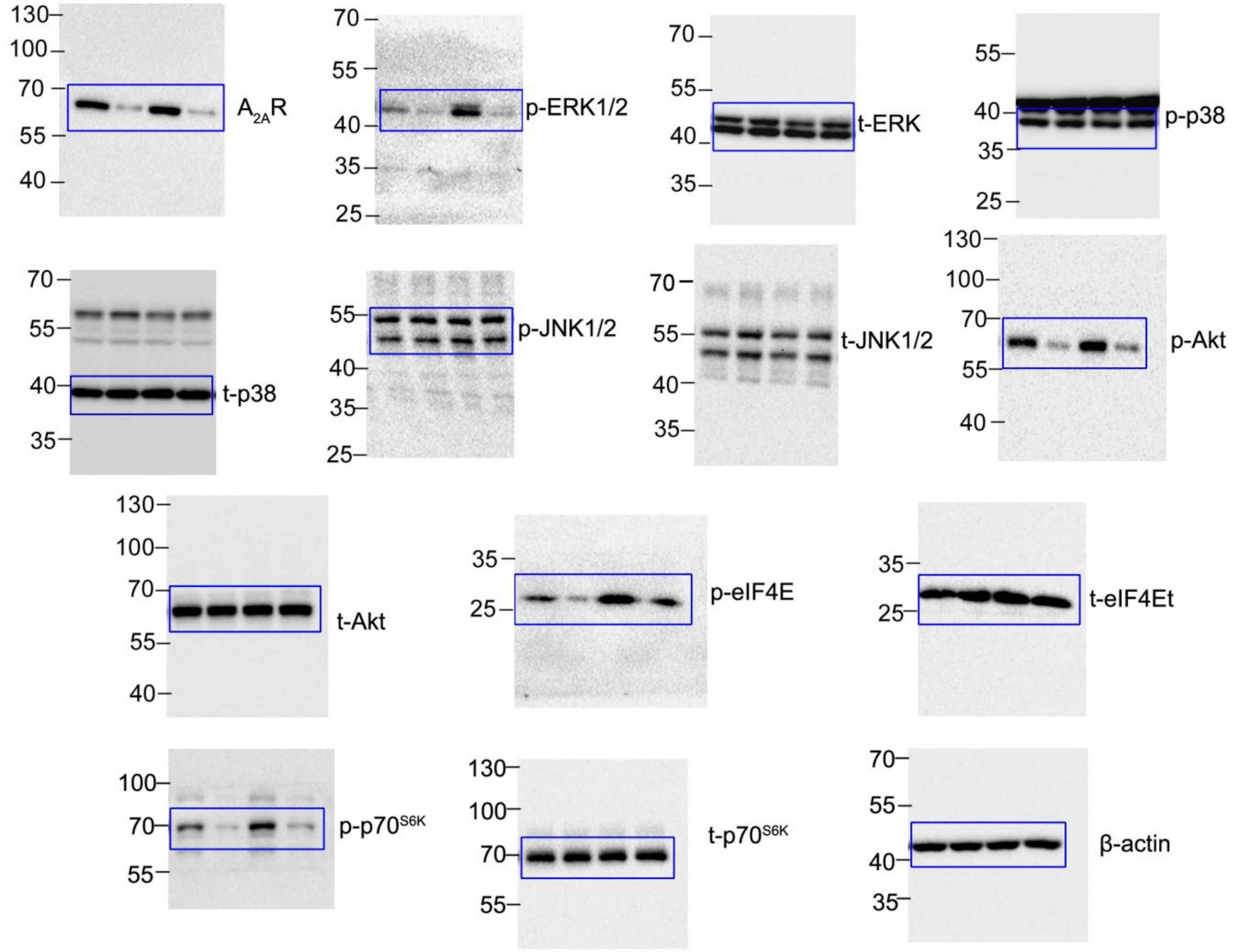

Figure 9f

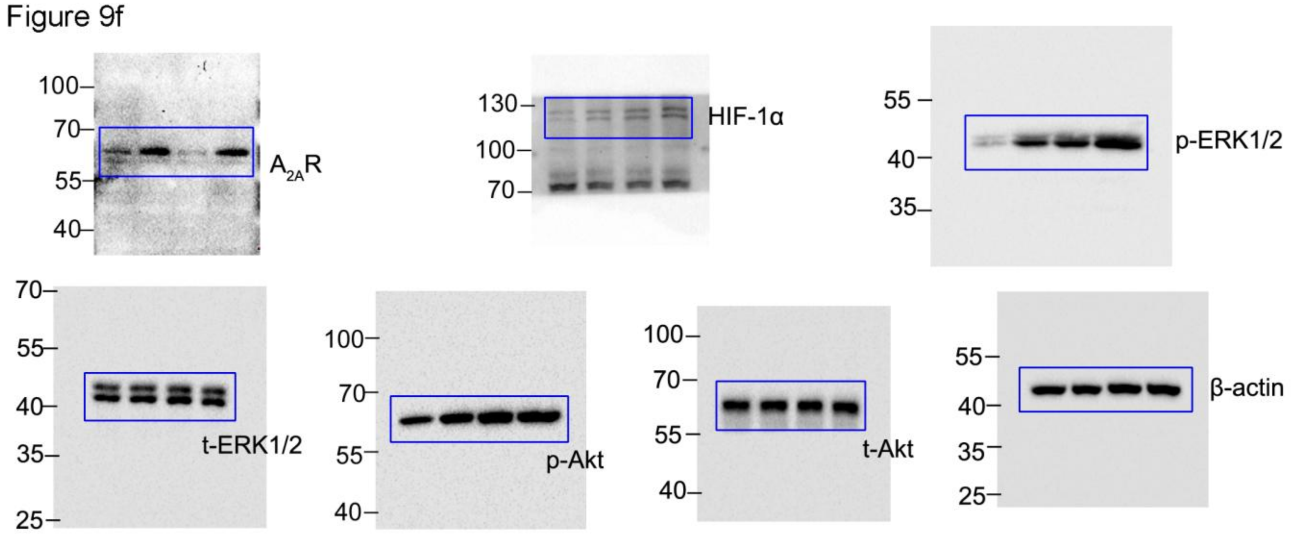

Supplementary Figure 17. Uncropped scans of the Western blots presented in Fig. 9e and 9f.

Figure 9g

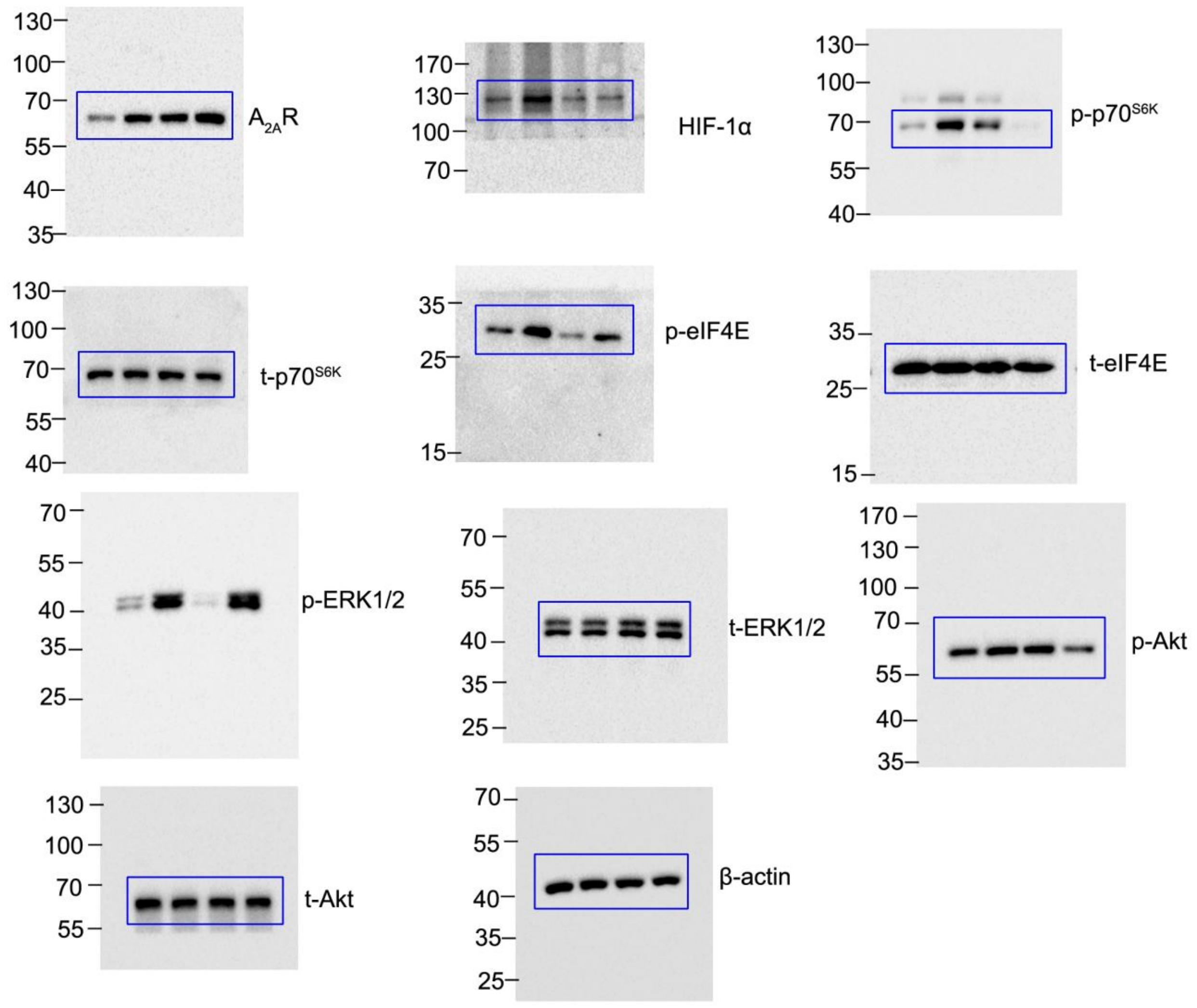

Supplementary Figure 18. Uncropped scans of the Western blots presented in Fig. 9g.

Target gene	Sequence
human ADORA1	F: 5'- TGCGAGTTCGAGAAGGTCATC-3'
	R: 5'- AGCTGCTTGCGGATTAGGTA-3'
human ADORA2A	F: 5'- CGAGGGCTAAGGGCATCATTG-3'
	R: 5'- CTCCTTTGGCTGACCGCAGTT-3'
human ADORA2B	F: 5'- CTCTTCCTCGCCTGCTTCGTG-3'
	R: 5'- TTATACCTGAGCGGGACACAG-3'
human ADORA3	F: 5'- TACATCATTCGGAACAAACTC-3'
	R: 5'- GTCTTGAACTCCCGTCCATAA-3'
human HIF-1α	F: 5'- GAACGTCGAAAAGAAAAGTCTCG -3'
	R: 5'- CCTTATCAAGATGCGAACTCACA -3'
human HIF-2α	F: 5'- CGGAGGTGTTCTATGAGCTGG-3'
	R: 5' - AGCTTGTGTGTTCGCAGGAA-3'
human GLUT1	F: 5'- GGCCAAGAGTGTGCTAAAGAA-3'
	R: 5'- ACAGCGTTGATGCCAGACAG -3'
human HK1	F: 5'- GCTCTCCGATGAAACTCTCATAG-3'
	R: 5'- GGACCTTACGAATGTTGGCAA-3'
human GPI	F: 5'- CAAGGACCGCTTCAACCACTT-3'
	R: 5'- CCAGGATGGGTGTGTTTGACC-3'
human PFKFB3	F: 5'- CTCGCATCAACAGCTTTGAGG-3'
	R: 5'- TCAGTGTTTCCTGGAGGAGTC-3'
human PFKP	F:5'- GCATGGGTATCTACGTGGGG-3'
	R:5'- CTCTGCGATGTTTGAGCCTC-3'

human ALDOA	F:5'- ATGCCCTACCAATATCCAGCA-3'
	R:5'- GCTCCCAGTGGACTCATCTG-3'
human GAPDH	F:5'- GGATTTGGTCGTATTGGG-3'
	R:5'- GGAAGATGGTGATGGGATT-3'
human PGK1	F:5'- TGGACGTTAAAGGGAAGCGG-3'
	R:5'- GCTCATAAGGACTACCGACTTGG-3'
human ENO1	F:5'- AAAGCTGGTGCCGTTGAGAA-3'
	R:5'- GGTTGTGGTAAACCTCTGCTC-3'
human PDK1	F:5'-CTGTGATACGGATCAGAAACCG-3'
	R:5'- TCCACCAAACAATAAAGAGTGCT-3'
human LDHA	F:5'- ATGGCAACTCTAAAGGATCAGC-3'
	R:5'- CCAACCCCAACAACTGTAATCT-3'
human LDHB	F:5'- TGGTATGGCGTGTGCTATCAG-3'
	R:5'- TTGGCGGTCACAGAATAATCTTT-3'
human NOTCH1	F:5'- GAGGCGTGGCAGACTATGC -3'
	R:5'- CTTGTACTCCGTCAGCGTGA -3'
human NOTCH2	F:5'- CAACCGCAATGGAGGCTATG -3'
	R:5'- GCGAAGGCACAATCATCAATGTT -3'
human NOTCH3	F:5'- TGGCGACCTCACTTACGACT-3'
	R:5'- CACTGGCAGTTATAGGTGTTGAC-3'
human CXCR4	F:5'- ACTACACCGAGGAAATGGGCT-3'
	R:5'- CCCACAATGCCAGTTAAGAAGA -3'
human CD34	F:5'- CTACAACACCTAGTACCCTTGGA-3'

	R:5'- GGTGAACACTGTGCTGATTACA-3'
human PIGF	F:5'-TGACATGGTTGTGCATCTGTT-3'
	R:5'- ACTCTATCAGTGGTGCTCCATAC-3'
human VEGFA	F:5'- CCC ACT GAG GAG TCC AAC AT-3'
	R:5'-TTT CTT GCG CTT TCG TTT TT-3'
human ANGPT2	F:5'- AACTTTCGGAAGAGCATGGAC-3'
	R:5'- CGAGTCATCGTATTCGAGCGG-3'
human APLN	F:5'- GTCTCCTCCATAGATTGGTCTGC-3'
	R:5'- GGAATCATCCAAACTACAGCCAG-3'
human HEY1	F:5'- GTTCGGCTCTAGGTTCCATGT-3'
	R:5'- CGTCGGCGCTTCTCAATTATTC -3'
human HEY2	F:5'- AAGGCGTCGGGATCGGATAA-3'
	R:5'- AGAGCGTGTGCGTCAAAGTAG-3'
Human NTN4	F:5'- GAGTAGCTGGAGTGAGTTCCC-3'
	R:5'- TCTGCCCAGAGTTTTCGCC-3'
human DLL1	F: 5'- GATTCTCCTGATGACCTCGCA-3'
	R: 5'- TCCGTAGTAGTGTTCGTCACA-3'
human DLL4	F: 5'- GTCTCCACGCCGGTATTGG-3'
	R: 5'- CAGGTGAAATTGAAGGGCAGT-3'
human HES1	F: 5'- TCAACACGACACCGGATAAAC-3'
	R: 5' - GCCGCGAGCTATCTTTCTTCA -3'
human JAG1	F: 5'- GTCCATGCAGAACGTGAACG -3'
	R: 5'- GCGGGACTGATACTCCTTGA -3'

human NPARP	F: 5'- TCAACGTGAACTCGTTCGGG-3'
	R: 5'- ACTTCGCCTTGGTGATGAGAT-3'
human NRP2	F: 5'- GCTGGCTATATCACCTCTCCC -3'
	R: 5'- TCTCGATTTCAAAGTGAGGGTTG -3'
human GUSB	F: 5'- TGACCGCTATGGGATTGT -3'
	R: 5'-AGCCTTGGGTGCTACTGG-3'
18S ribosomal RNA	F: 5'- CTTAGAGGGACAAGTGGCG-3'
	R: 5'- ACGCTGAGCCAGTCAGTGTA -3'
mouse ADORA2A	F: 5'- AGCAACCTGCAGAACGTCACAAAC-3'
	R: 5'- TGGCAATAGCCAAGAGGCTGAAGA-3'
mouse HIF-1α	F: 5'- TGCCCCAGATTCAAGATCAGC-3'
	R: 5'- GGCTGGGAAAAGTTAGGAGTGT-3'
mouse HIF-2α	F: 5'- GAGGAAGGAGAAATCCCGTGA-3'
	R: 5' - TATGTGTCCGAAGGAAGCTGA-3'
mouse GLUT1	F: 5'- GCAGTTCGGCTATAACACTGG-3'
	R: 5'- GCGGTGGTTCCATGTTTGATTG-3'
mouse HK1	F: 5'- AACGGCCTCCGTCAAGATG-3'
	R: 5'- GCCGAGATCCAGTGCAATG-3'
mouse GPI	F: 5'- CTCAAGCTGCGCGAACTTTTT -3'
	R: 5'- GGTTCTTGGAGTAGTCCACCAG-3'
mouse PFKFB3	F: 5'- GATCTGGGTGCCCGTCGATCACCG-3'
	R: 5'- CAGTTGAGGTAGCGAGTCAGCTTC-3'
mouse ALDOA	F:5'- AGTCCACCGGAAGCATTGC-3'

	R:5'- CAGCCCCTGGGTAGTTGTC-3'
mouse GAPDH	F:5'- AGGTCGGTGTGAACGGATTTG-3'
	R:5'- GGGGTCGTTGATGGCAACA-3'
mouse PGK1	F:5'- ATGTCGCTTTCCAACAAGCTG-3'
	R:5'- GCTCCATTGTCCAAGCAGAAT-3'
mouse ENO1	F:5'- TGCGTCCACTGGCATCTAC-3'
	R:5'- CAGAGCAGGCGCAATAGTTTTA-3'
mouse PDK1	F:5'- GGACTTCGGGTCAGTGAATGC-3'
	R:5'- TCCTGAGAAGATTGTCGGGGA-3'
mouse LDHA	F:5'- CAAAGACTACTGTGTAACTGCGA -3'
	R:5'- TGGACTGTACTTGACAATGTTGG-3'
mouse LDHB	F:5'- TGCGTCCGTTGCAGATGAT-3'
	R:5'- TTTCGGAGTCTGGAGGAACAA-3'
mouse HPRT	F:5'- AGTGTTGGATACAGGCCAGAC-3'
	R:5'- CGTGATTCAAATCCCTGAAGT-3'
mouse HMBS	F:5'- GAGTCTAGATGGCTCAGATAGCATGC-3'
	R:5'- CCTACAGACCAGTTAGCGCACATC-3'

F: forward primer; R: reverse primer

Supplementary Table 1

Primer sets used for Real-Time PCR
